# SmD1 Modulates the miRNA Pathway Independently of Its Pre-mRNA Splicing Function

**DOI:** 10.1371/journal.pgen.1005475

**Published:** 2015-08-26

**Authors:** Xiao-Peng Xiong, Georg Vogler, Krishna Kurthkoti, Anastasia Samsonova, Rui Zhou

**Affiliations:** 1 Tumor Initiation and Maintenance Program, Sanford-Burnham Medical Research Institute, La Jolla, California, United States of America; 2 Development, Aging and Regeneration Program, Sanford-Burnham Medical Research Institute, La Jolla, California, United States of America; 3 Department of Oncology, University of Oxford, Oxford, United Kingdom; University of California Berkeley, UNITED STATES

## Abstract

microRNAs (miRNAs) are a class of endogenous regulatory RNAs that play a key role in myriad biological processes. Upon transcription, primary miRNA transcripts are sequentially processed by Drosha and Dicer ribonucleases into ~22–24 nt miRNAs. Subsequently, miRNAs are incorporated into the RNA-induced silencing complexes (RISCs) that contain Argonaute (AGO) family proteins and guide RISC to target RNAs via complementary base pairing, leading to post-transcriptional gene silencing by a combination of translation inhibition and mRNA destabilization. Select pre-mRNA splicing factors have been implicated in small RNA-mediated gene silencing pathways in fission yeast, worms, flies and mammals, but the underlying molecular mechanisms are not well understood. Here, we show that SmD1, a core component of the *Drosophila* small nuclear ribonucleoprotein particle (snRNP) implicated in splicing, is required for miRNA biogenesis and function. SmD1 interacts with both the microprocessor component Pasha and pri-miRNAs, and is indispensable for optimal miRNA biogenesis. Depletion of SmD1 impairs the assembly and function of the miRISC without significantly affecting the expression of major canonical miRNA pathway components. Moreover, SmD1 physically and functionally associates with components of the miRISC, including AGO1 and GW182. Notably, miRNA defects resulting from SmD1 silencing can be uncoupled from defects in pre-mRNA splicing, and the miRNA and splicing machineries are physically and functionally distinct entities. Finally, photoactivatable-ribonucleoside-enhanced crosslinking and immunoprecipitation (PAR-CLIP) analysis identifies numerous SmD1-binding events across the transcriptome and reveals direct SmD1-miRNA interactions. Our study suggests that SmD1 plays a direct role in miRNA-mediated gene silencing independently of its pre-mRNA splicing activity and indicates that the dual roles of splicing factors in post-transcriptional gene regulation may be evolutionarily widespread.

## Author Summary

microRNAs (miRNAs) are a class of small regulatory RNAs that fine-tune gene expression by reducing protein output from their target messenger RNAs and are implicated in myriad physiological and pathological processes. miRNAs are generated from long primary transcripts via sequential actions of the Drosha/Pasha and Dicer ribonucleases. Mature miRNAs are incorporated into the miRISC effector complexes that contain AGO family member proteins and serve as specificity determinants to guide miRISCs to their target RNAs. Previous studies suggested that select proteins implicated in the processing of messenger RNAs are required for the miRNA production/function, but the underlying molecular mechanism is not well understood. Here we show that SmD1, an essential protein implicated in the processing of messenger RNAs, directly interacts with both Pasha and primary miRNA transcripts and is required for optimal miRNA production. Furthermore, SmD1 associates with multiple components of the miRNA effector machinery and is required for miRNA function. Finally, our analysis reveals that defects in the miRNA pathway can be uncoupled from those in messenger RNA processing, and that the miRNA biogenesis and messenger RNA processing machineries are physically and functionally distinct entities. Our data thus suggests that SmD1 modulates the miRNA pathway independent of its role in messenger RNA processing.

## Introduction

miRNAs are a class of ~22–24 nt endogenous regulatory RNAs present in all cell types of multicellular organisms [1,2]. By regulating the expression of diverse target RNAs, miRNAs play a key role in myriad biological processes including development, homeostasis, and innate immunity. In *Drosophila*, canonical miRNA biogenesis starts with RNA polymerase II-mediated transcription of long stem-loop primary miRNA transcripts (pri-miRNAs). They are processed in the nucleus by the Drosha/Pasha ribonuclease III (RNase III) microprocessor complex into ~60–70 nt precursor miRNAs (pre-miRNAs), via a reaction referred to as cropping [3,4,5]. Precursor miRNAs are subsequently exported to the cytoplasm by Exportin5/Ran-GTP and further processed in a dicing reaction by a second RNase III complex, the Dicer 1 (Dcr-1)/Loqs-PB complex, thereby liberating ~22–24 nt miRNA duplexes [6,7,8,9,10,11,12]. The mature miRNA strand of the duplex is predominantly incorporated into Argonaute 1 (AGO1)-containing miRNA-induced silencing complexes (miRISC). miRISC in turn engages target mRNAs via complementary base pairing between the seed region of miRNAs (positions 2–8) and miRNA-binding sites (primarily in the 3’ UTR of target mRNAs), and represses gene expression post-transcriptionally by promoting target mRNA destabilization and/or translation inhibition [13,14,15,16,17,18].

Over a decade of investigation has identified a collection of core components of the miRNA biogenesis and functional machineries, and delineated the framework of the molecular mechanism underlying the miRNA pathway. However, our knowledge of the miRNA pathways is far from complete, and many accessory factors that modulate miRNA biology await identification and functional characterization. A number of recent studies highlight extensive crosstalk between pre-mRNA splicing and small RNA-mediated gene silencing pathways. For example, the core RISC component AGO2 has been implicated in modulating pre-mRNA splicing both in mammals and in *Drosophila* [19,20]. Conversely, select splicing factors have been shown to impact small RNA-mediated gene silencing pathways. It has been reported that mutations in genes encoding a subset of splicing factors compromise RNAi in the fission yeast *Schizosaccharomyces pombe* [21], and that in plants, mutations in splicing factor genes compromise small RNA biogenesis [22]. In addition, the multifunctional human RNA-binding protein hnRNP A1, which regulates alternative splicing, impacts the processing of *miR-18a* and *let-7a* [23,24]. Furthermore, the KH-type splicing regulatory protein (KSRP) has been shown to positively regulate the biogenesis of *miR-155* and *let-7* [25,26]. Moreover, genome-wide RNAi screens conducted in *C*. *elegans* and cultured *Drosophila* cells show that depletion of certain splicing factors compromises RNAi [27,28,29]. Finally, a recent analysis of phylogenetic conservation of candidate RNAi factors suggests that select splicing factors are required for small RNA-mediated gene silencing [30].

SmD1, together with six other small ribonucleoprotein particle (snRNP) proteins (SmB, SmD2, SmD3, SmE, SmF and SmG), form a heptameric ring structure surrounding the U-rich small nuclear RNAs (snRNAs) [31]. These snRNP proteins constitute core components of the snRNP and play a key role in pre-mRNA splicing [32]. We recently showed that SmD1 depletion in cultured *Drosophila* cells compromises small interfering RNA (siRNA) biogenesis and function independently of its role in pre-mRNA splicing [33]. In the current study, we investigate the role of SmD1 in the miRNA pathway. We find that SmD1 depletion leads to a reduction in levels of mature miRNAs, which is accompanied by a derepression of the corresponding target messenger RNAs and a concomitant accumulation of primary miRNA transcripts. In addition, SmD1 associates with the microprocessor component Pasha and is required for optimal microprocessor activity. In contrast, SmD1 is dispensable for Dicer-mediated processing of pre-miRNAs into mature miRNAs. Furthermore, our analysis reveals that SmD1 is required for miRNA function besides its role in miRNA biogenesis. Specifically, SmD1 associates with the miRISC components AGO1 and GW182, and is required for optimal miRISC function. Moreover, we show that select splicing factors such as SmD1, but not pre-mRNA splicing per se, modulate the miRNA pathway, as defects in miRNA biogenesis and in pre-mRNA splicing can be uncoupled, and that SmD1 inactivation does not affect the expression of canonical miRNA pathway components. Finally, PAR-CLIP analysis identifies numerous SmD1-binding events across the transcriptome and reveals direct SmD1-miRNA interactions. Taken together, our study identifies SmD1 as a new modulator of the miRNA pathway at multiple levels, and provides direct evidence to support and extend the notion that select splicing factors are critical modulators of small RNA pathways in complex multicellular organisms, beyond the context of the spliceosome.

## Results

### SmD1 is required for miRNA biogenesis and/or stability

**Fig 1:**
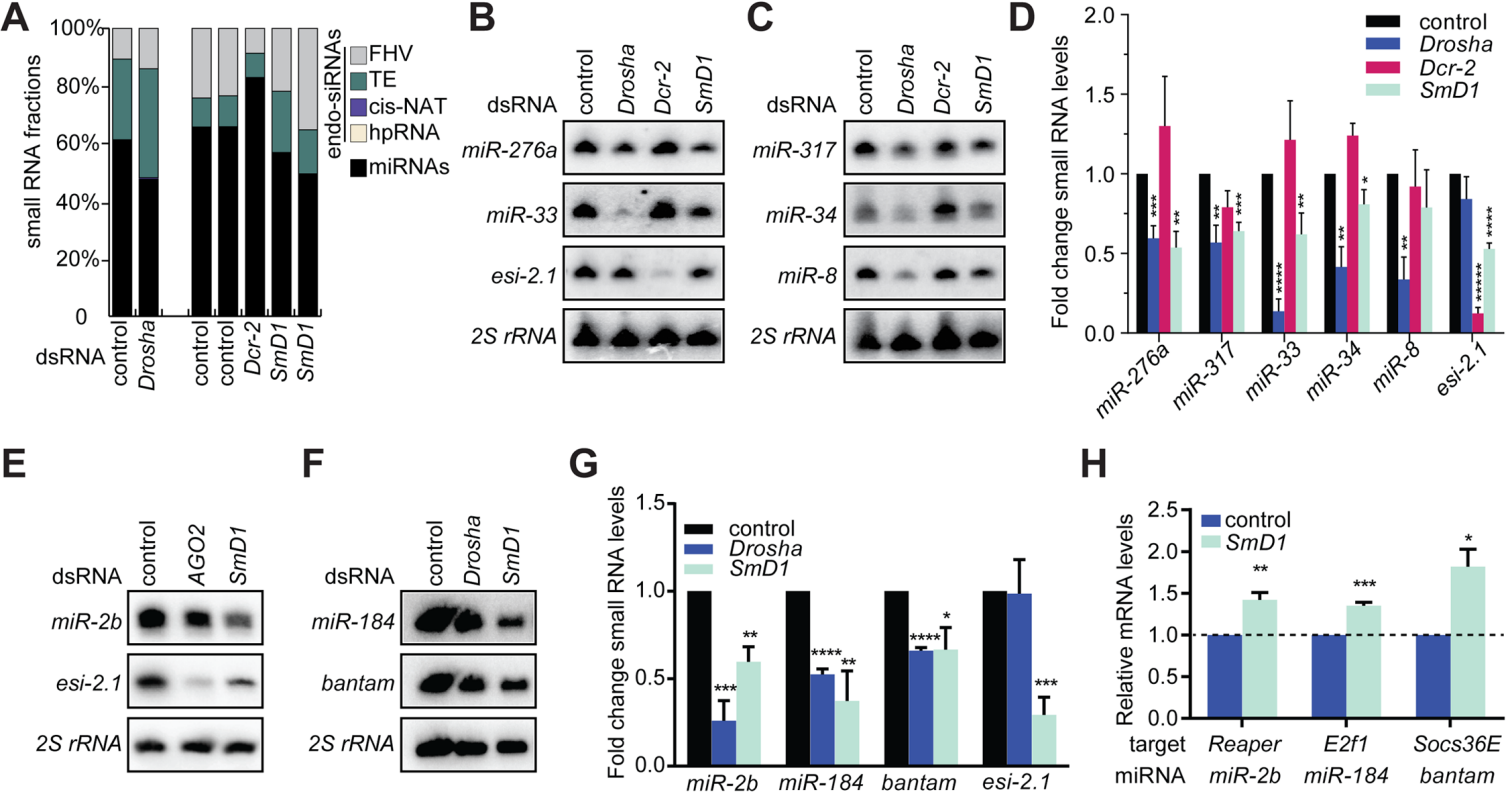
Loss of SmD1 compromises miRNA biogenesis. (**A**) small RNA samples from various dsRNA-treated S2 cells were subject to deep sequencing. The proportions of various classes of endogenous siRNAs and miRNAs in each library are shown. Note that small RNA libraries were prepared in two batches (batch 1: control and *Drosha* knockdown; batch 2: two independent control samples, one *Dcr-2* knockdown sample, and two independent *SmD1* knockdown samples). Unless noted otherwise in this manuscript, dsRNA against the firefly luciferase gene serve as a control. (**B,C,E,F**) S2 cells were treated with various dsRNAs (above) and levels of the endogenous siRNA *esi-2*.*1*, various miRNAs or *2S* rRNA (loading control) were measured by Northern blot. (**D,G**) Quantification of miRNA and *esi-2*.*1* levels (n = 3) normalized against *2S* rRNA levels and compared with controls. Unless noted otherwise, data in this manuscript are shown as mean + SD. *p < 0.05, **p < 0.01, ***p < 0.001, ****p < 0.0001. (**H**) Levels of various mRNA targets of the corresponding miRNAs (below) in SmD1-depleted cells or control cells were measured by RT-qPCR and normalized against the control *rp49* mRNA (n ≥ 3).

We first examined small RNA expression profiles in control S2 cells or cells depleted of Drosha, Dcr-2 or SmD1 (Fig 1A and S1 Table). As expected, compared with control samples, depletion of Drosha led to a reduction in the proportion of miRNAs in the total small RNA population. As a consequence, the proportions of all classes of endogenous siRNAs expanded, including those derived from the Flock House Virus (FHV), transposable element (TE), convergently transcribed RNAs (cis-NAT) and hpRNAs. In contrast, depletion of the siRNA biogenesis enzyme Dcr-2 caused the opposite phenotype. Notably, *SmD1* knockdown led to a shrinkage of the miRNA population and an artificial expansion of endo-siRNAs. Considering our previous findings showing that SmD1 is required for siRNA biogenesis and SmD1 depletion led to a reduction in levels of endo-siRNAs [33], our data strongly suggest that SmD1 depletion can cause a comparable or even stronger degree of reduction in levels of miRNAs than in levels of siRNAs. To test this directly, we measured levels of endogenous siRNAs and miRNAs by Northern blot in *SmD1* knockdown cells. Consistent with our recent finding [33], SmD1 depletion led to a marked decrease in levels of the endogenous siRNA *esi-2*.*1* (Fig 1B and 1E). Importantly, levels of several miRNAs, including *miR-33*, *miR-34*, *miR-276a*, *miR-317*, *miR-2b*, *miR-184* and *miR-bantam*, were significantly reduced upon *SmD1* knockdown (Fig 1B–1G), reminiscent of the phenotype elicited by the loss of the canonical miRNA biogenesis enzyme Drosha. As a negative control, depleting canonical siRNA pathway components such as Dcr-2 or AGO2 predominantly affected levels of *esi-2*.*1*, but not miRNAs (Fig 1B–1E). To rule out potential off-target effects associated with dsRNAs, we tested an independent dsRNA against *SmD1* and observed a similar impact on small RNA levels (S1 Fig). We also detected a significant increase in steady-state levels of a subset of target mRNAs for several miRNAs implicated in cell proliferation and apoptosis (*Reaper*, *E2f1* and *Socs36E* as targets for *miR-2b*, *miR-184* and *miR-bantam*, respectively) (Fig 1H) [34,35], consistent with the notion that miRNAs repress target gene expression by inhibiting mRNA translation and promoting target mRNA decay. We conclude that SmD1 is required for optimal miRNA biogenesis and/or stability.

Besides the afore-mentioned miRNAs, which are constitutively expressed in S2 cells, we also examined levels of the miRNA *let-7*, which is not expressed in naïve S2 cells but becomes highly induced upon treatment with 20-hydroxyecdysone (20-E) [36]. We detected lower levels of *let-7* in SmD1-depleted cells (S2 Fig). These data reinforce the notion that SmD1 is required for optimal miRNA biogenesis. *let-7* is highly expressed in the *Drosophila* heart, and is required for *Drosophila* heart function. Interestingly, depletion of SmD1 specifically in cardiac cells of adult *Drosophila* causes several defects in cardiac function (S3 Fig). These observations suggest that the cardiac phenotype elicited by SmD1 depletion is linked, at least in part, to defects in *let-7* biogenesis.

### SmD1 is selectively required for the processing of pri-miRNAs into pre-miRNAs

**Fig 2:**
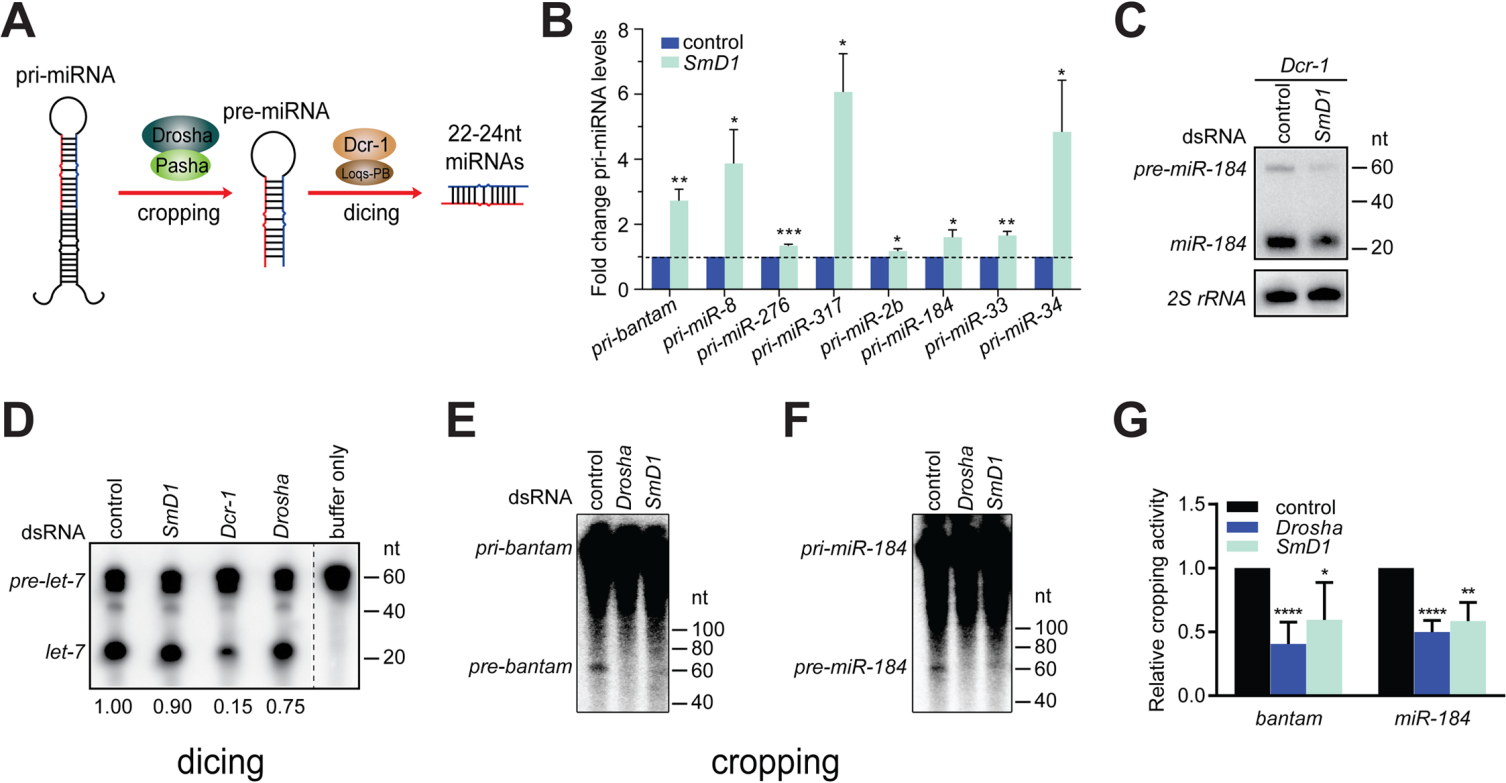
Depletion of SmD1 compromises microprocessor activity. (**A**) A schematic of the cropping and dicing steps of miRNA biogenesis. (**B**) Levels of various primary miRNA transcripts in SmD1-depleted cells or control cells were measured by RT-qPCR and normalized against the control *rp49* mRNA (n ≥ 3). (**C**) S2 cells were treated with control or *SmD1* dsRNAs together with the *Dcr-1* dsRNA, and levels of the precursor and mature *miR-184* were measured by Northern blot. (**D**) Cytoplasmic lysates from S2 cells treated various dsRNAs were incubated with radiolabeled *pre-let-7* substrate to generate mature *let-7*. RNAs were extracted and resolved by denaturing urea-PAGE. Quantification of Dcr-1 activity is shown at the bottom. (**E,F**) Total lysates from S2 cells treated various dsRNAs were incubated with radiolabeled *pri-bantam* (**E**) or *pri-miR-184* (**F**) to generate pre-miRNAs. (**G**) The microprocessor activity, represented as the amount of resultant pre-miRNAs, was quantified and normalized to controls (n ≥ 4).

miRNA biogenesis consists of multiple steps, including Drosha-mediated processing of pri-miRNAs into pre-miRNAs and Dcr-1-mediated conversion of pre-miRNAs into mature miRNAs, referred to as cropping and dicing, respectively (Fig 2A). To further define the biochemical step(s) of miRNA biogenesis that require SmD1, we examined the impact of *SmD1* inactivation on levels of pri-miRNAs and pre-miRNAs. As expected, we detected a marked accumulation of several pri-miRNAs in S2 cells depleted of the microprocessor component Drosha (S4 Fig). Importantly, SmD1 phenocopies Drosha, albeit to a moderate extent (Fig 2B), suggesting that SmD1 is required for microprocessor-mediated processing of pri-miRNAs. To assess whether SmD1 is required for Dcr-1-mediated processing of pre-miRNAs into mature miRNAs, we treated S2 cells with dsRNAs targeting the firefly luciferase (as control) or *SmD1*, together with dsRNAs targeting *Dcr-1* (to facilitate the detection of pre-miRNAs), and performed Northern blot to measure levels of pre-miRNAs. We found that compared to control samples, loss of *SmD1* caused a reduction in levels of *pre-miR-184* (Fig 2C). These observations strongly suggest that SmD1 is dispensable for Dcr-1-mediated processing of pre-miRNAs into mature miRNAs, as otherwise we would have detected an accumulation of pre-miRNAs upon *SmD1* inactivation. We note that it remains formally possible that SmD1 is required for the processing of pre-miRNAs into mature miRNAs, and the observed decrease in the overall pre-miRNA levels upon *SmD1* knockdown is an accumulative effect of impaired microprocessor activity (leading to less pre-miRNA production) and less efficient Dcr-1-mediated pre-miRNA processing (leading to accumulation of pre-miRNAs). Next, to definitively elucidate the requirement for SmD1 in different steps of miRNA biogenesis, we prepared total or cytoplasmic lysates from S2 cells treated with various dsRNAs, incubated lysates with radiolabeled pri-miRNAs or pre-miRNAs, and measured the microprocessor or Dcr-1 activities by monitoring the production of pre-miRNAs or mature miRNAs, respectively. Our analysis revealed that *SmD1*-deficient cell lysate is as competent as the control lysate in carrying out Dcr-1-mediated processing of pre-miRNAs into mature miRNAs (Fig 2D). In contrast, *SmD1*-deficient cell lysate displays significant defects in Drosha-mediated conversion of pri-miRNAs into pre-miRNAs (Figs 2E–2G and S5). These data demonstrate that SmD1 is specifically required for cropping, but is dispensable not the dicing step during miRNA biogenesis.

### SmD1 interacts with the microprocessor component Pasha

**Fig 3:**
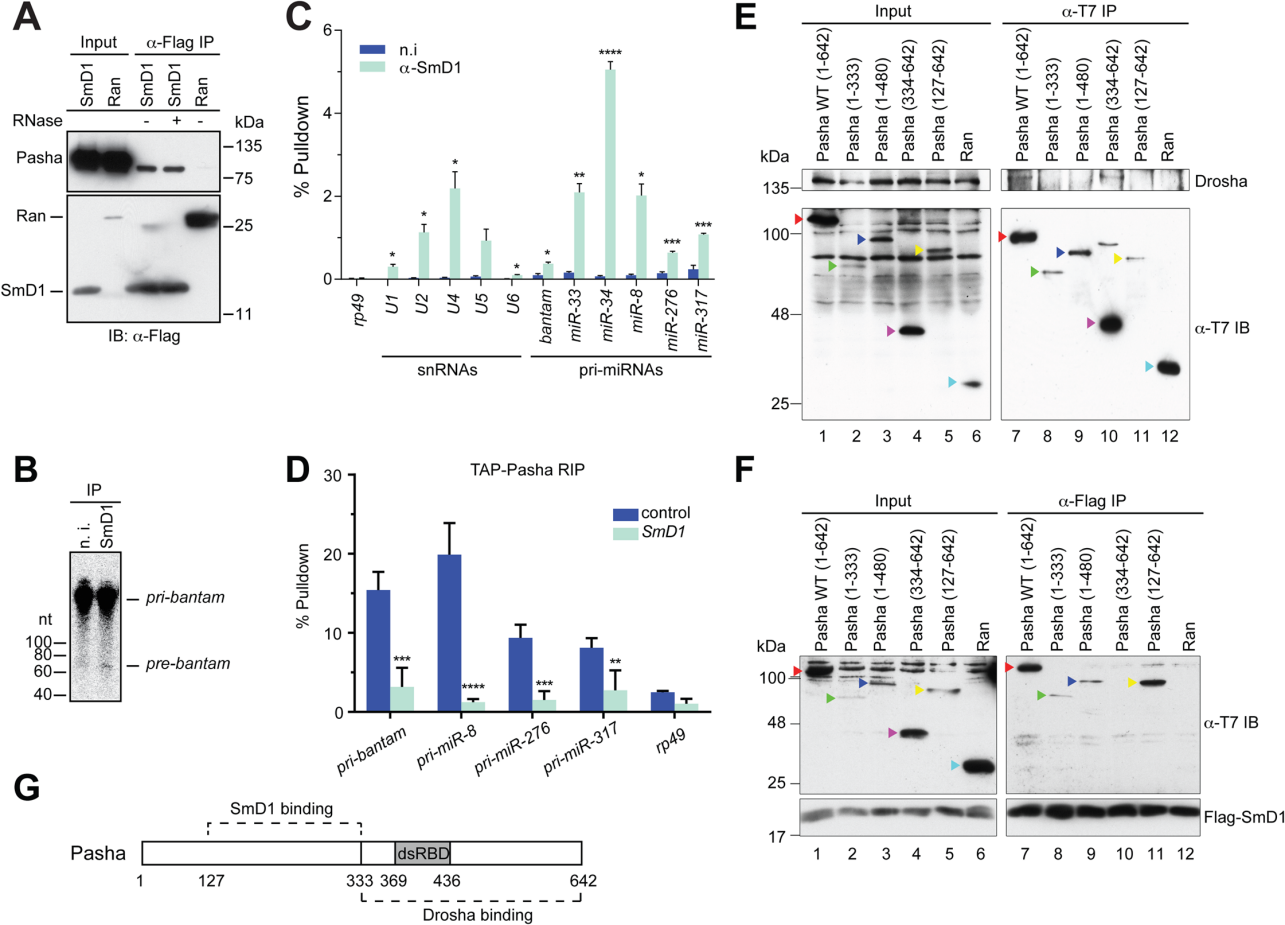
SmD1 interacts with the microprocessor component Pasha and pri-miRNAs. (**A**) Lysates from S2 cells expressing Flag-tagged SmD1 or the control protein Ran were immunoprecipitated with anti-Flag antibody. Input and immunoprecipitated material (treated with or without RNase A) were subject to immunoblotting using various antibodies (left). (**B**) S2 cell lysates were subject to immunoprecipitation using anti-SmD1 antibody or the control non-immune (n.i.) serum. Microprocessor activity associated with the isolated complexes was assessed using *pri-miR-184* as substrate. (**C**) Total RNA was extracted from immunopurified SmD1 complexes or control samples (n.i.) and subject to RT-qPCR to measure levels of various snRNAs, pri-miRNAs or the control mRNA *rp49* (mean + SEM, n ≥ 3). Percentage of pulldown relative to the input is shown. (**D**) S2 cells stably expressing TAP-Pasha were treated with control or *SmD1* dsRNAs. Relative enrichment of various pri-miRNAs in purified TAP-Pasha complexes was measured by RT-qPCR (n = 4). (**E,F**) Various T7-tagged full length or truncated Pasha proteins or the control protein Ran were either expressed individually (**E**) or co-expressed with Flag-SmD1 (**F**) in S2 cells. Cell lysates were subject to immunoprecipitation using either anti-T7 (**E**) or anti-Flag antibodies (**F**) and the isolated complexes were analyzed by immunoblotting with anti-Drosha (**E**), anti-Flag (**F**) and anti-T7 (**E,F**) antibodies. Various Pasha proteins are labeled by color-coded arrowheads. (**G**) A schematic of Pasha showing Drosha- and SmD1-binding domains. dsRBD represents dsRNA-binding domain.

Having shown that SmD1 is required for optimal microprocessor activity, we next examined whether SmD1 associates with components of the microprocessor. Consistent with the requirement for SmD1 in the cropping step, we found that at Flag-tagged SmD1, but not the control protein Ran, co-immunoprecipitated with the endogenous microprocessor component Pasha (Fig 3A). In addition, the recovery of Pasha in the SmD1 complex is resistant to RNase A treatment, indicating RNA-independent protein-protein interactions. It remains to be determined whether the observed SmD1-Pasha interaction is direct or through protein intermediates. While we were unable to detect endogenous Drosha in the SmD1 complex by immunoblotting, most likely due to the low sensitivity of the Drosha antibody, moderate but clearly above background levels of microprocessor activity can be recovered from immunopurified endogenous SmD1 complex (Fig 3B). These observations underscore the functional relevance of the observed SmD1-Pasha interaction. Next, we performed RNA immunoprecipitation (**RIP**) assays to assess whether SmD1 could associate with pri-miRNAs, the substrates for the microprocessor. Our analysis revealed significant enrichment in the SmD1 complex all six pri-miRNAs that we have examined, the degree of which matched or even exceeded that of cognate SmD1-binding small nuclear RNAs (Fig 3C). In contrast, no significant enrichment of the control mRNA *rp49* was observed. The observed interactions of SmD1 with Pasha and pri-miRNAs suggest that SmD1 might modulate miRNA biogenesis by serving as a molecular bridge to facilitate the recognition of pri-miRNAs by the microprocessor. To test this possibility, we performed RIP assay using a stable cell line expressing TAP-tagged Pasha and detected a significant enrichment of pri-miRNAs in immunopurified TAP-Pasha complex (S6 Fig). Importantly, recovery of pri-miRNAs in the Pasha complex was severely blunted upon depletion of SmD1 (Fig 3D). Interestingly, we also observed a moderate degree of enrichment of the control mRNA *rp49* in the Pasha complex. This is consistent with the notion that the microprocessor complex potentially associates with and regulates the expression of a number of cellular mRNAs (S6 Fig) [37,38]. However, the association of the *rp49* mRNA with Pasha seems to be largely unaffected by SmD1 depletion (Figs S6 and 3D).

To investigate the molecular detail of the SmD1-Pasha interaction, we next sought to map the protein domains responsible for this interaction. We generated a series of T7-tagged full length and truncated Pasha proteins and examined their capability of interacting with Drosha and SmD1 by performing co-immunoprecipitation assays. As expected, we found that full length Pasha was able to co-immunoprecipitate with endogenous Drosha in cultured *Drosophila* S2 cells (Fig 3E, lane 7). In addition, the C-terminal fragment of Pasha (334–642) is also capable of pulling down Drosha (Fig 3E, lane 10). Our data are consistent with previous reports showing that the C-terminal fragment of DGCR8 interacts with Drosha in mammals [39,40]. Note that T7-tagged Pasha_127–642_ was unable to co-immunoprecipitate with endogenous Drosha, possibly due to inefficient folding and/or presentation of the T7 epitope in native T7-Pasha_127–642_ protein in total cell lysate, as T7-Pasha_127–642_ was poorly recovered in the anti-T7 immunoprecipitate (Fig 3E, lane 11, lower panel). In contrast, T7-Pasha_127–642_ was readily detectable in input samples, most likely because the T7 epitope can be efficiently recognized by the anti-T7 antibody in denatured T7-Pasha_127–642_ (Fig 3E, lane 5, lower panel). Next, we examined whether various truncated Pasha proteins can co-immunoprecipitate with SmD1. We found that Flag-tagged SmD1 was able to pull down both full length and a number of truncated Pasha mutants containing the region spanning amino acids 127–333 (Fig 3F, lanes 7–9, 11). Thus, it appears that distinct regions of Pasha are required for the Pasha-Drosha and Pasha-SmD1 interactions (Fig 3G). The stoichiometry of the SmD1-microprocessor complex is currently not clear, and it remains to be determined whether the observed Drosha-Pasha and SmD1-Pasha interactions are mutually exclusive, or all three proteins are present in the same complex. Collectively, these data demonstrate that SmD1 associates with both components of the miRNA biogenesis machinery and primary miRNA transcripts, and that SmD1 is required for optimal recognition of the pri-miRNAs by the microprocessor.

### SmD1 is required for miRNA function

**Fig 4:**
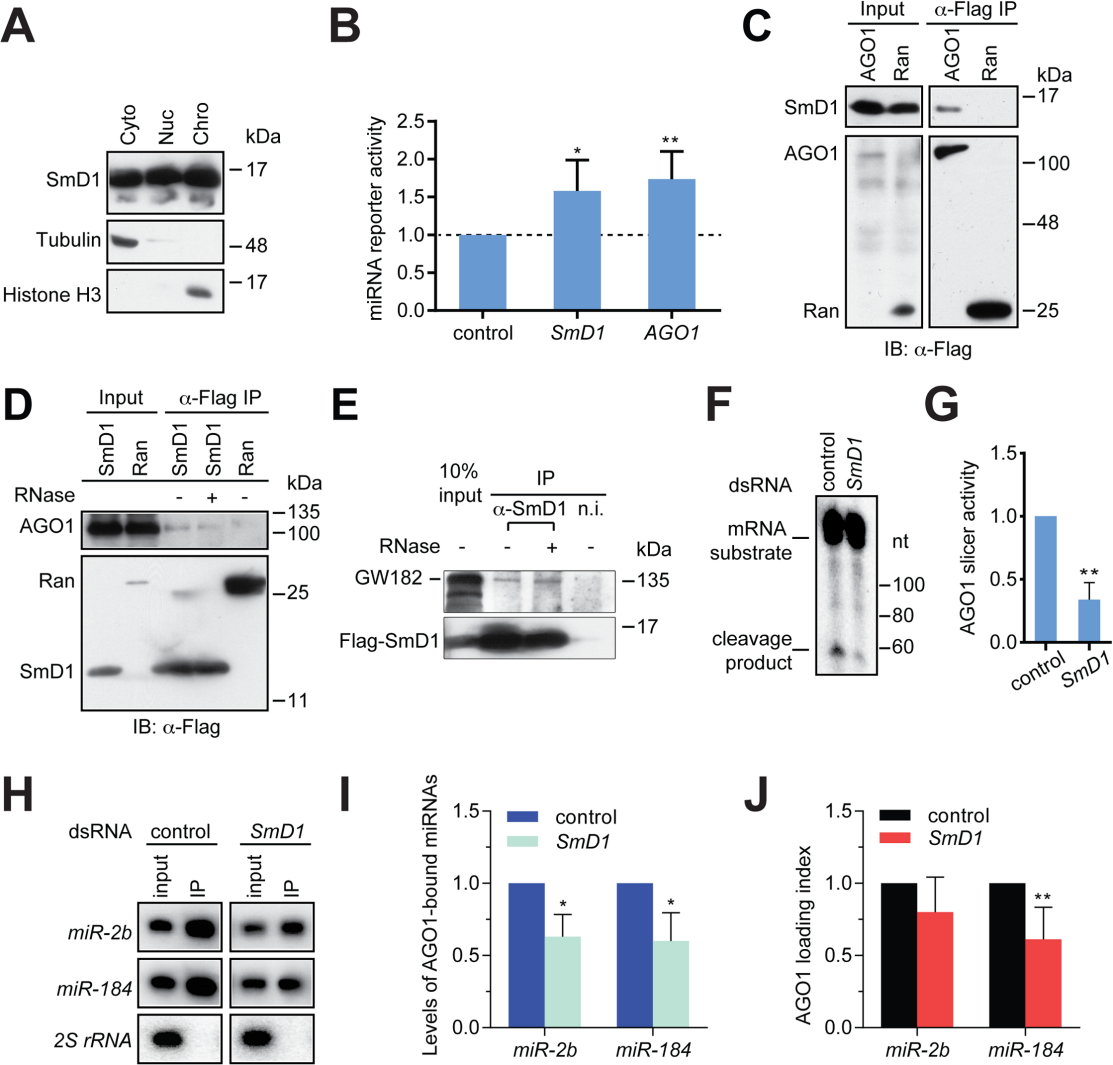
SmD1 interacts with miRISC components and loss of SmD1 abolishes miRISC assembly/function. (**A**) Cytoplasmic (Cyto), nuclear (Nuc) and chromatin (Chro) fractions from S2 cells were probed with antibodies against SmD1, Tubulin and Histone H3. (**B**) Expression construct for a *Renilla* luciferase reporter mRNA that carries 8 copies of *let-7* binding sites and a firefly luciferase (normalization control) were transfected into dsRNA treated cells (bottom) together with a synthetic *let-7* miRNA duplex. Depleting AGO1 or SmD1 led to a de-repression of the *let-7* reporter compared to control knockdown with *LacZ* dsRNA (n = 4). (**C**) Lysates from S2 cells expressing Flag-tagged AGO1 or the control protein Ran were immunoprecipitated with anti-Flag antibody. Input and immunoprecipitated material were subject to immunoblotting using anti-SmD1 and anti-Flag antibodies. (**D**) Lysates from cells expressing Flag-SmD1 or the control protein Ran were subject to immunoprecipitation using anti-Flag antibody. Input samples and immunoprecipitated material (treated with or without RNase A) were subject to immunoblotting using anti-AGO1 and anti-Flag antibodies. Note the anti-Flag panel in **D** is identical to that shown in **3A**. (**E**) Lysates from S2 cells expressing Flag-tagged SmD1 were immunoprecipitated with anti-SmD1 antibody or the non-immune serum. Input and immunoprecipitated material (treated with or without RNase A) were subject to immunoblotting using anti-GW182 and anti-Flag antibodies. (**F**) Minimal miRISC was immunopurified from TAP-AGO1 cell lysates using IgG agarose beads. Purified minimal miRISC was subsequently incubated with lysates from control or *SmD1*-knockdown cells in the presence of *let-7* miRNA duplex. The beads were washed thoroughly and the slicer activity of resultant miRISC was measured as cleavage of the cap-radiolabeled target mRNA that contains a perfectly complementary *let-7* site. (**G**) Quantification of the AGO1-miRISC slicer activity (n = 3). (**H**) TAP-AGO1 expressing cells were treated either with a control dsRNA against the firefly luciferase gene, or that against *SmD1*. Total RNA was extracted from either input cell lysates or immunopurified TAP-AGO1 complexes and subject to Northern blotting to detect various small RNAs (left). (**I**) Quantification of levels of AGO1-bound miRNAs (n = 3). (**J**) Quantification of AGO1-loading index for individual miRNAs by normalizing levels of AGO1-bound miRNAs against those in the input (n = 5).

Several lines of evidence point to a possible role of SmD1 in the effector phase of the miRNA pathway (i.e., miRISC assembly and function) besides its involvement in miRNA biogenesis: 1) SmD1 modulates siRISC assembly and function, and associates with several siRISC components, including AGO2 [33]; 2) SmD1, but not the control protein Ran, co-immunoprecipitates with *miR-2b* [33]; 3) in mammals SNRPD1 (ortholog of *Drosophila* SmD1) and AGO2 (a canonical miRISC component) interact with each other [33]; and 4) a considerable fraction of SmD1 is present in the cytoplasm (Fig 4A), where miRISC assembly and function primarily take place. To examine this possibility, we transfected SmD1-depleted S2 cells with a synthetic *let-7* miRNA duplex together with a *Renilla* luciferase reporter construct carrying 8 copies of imperfect *let-7*-binding sites in the 3’ UTR. A firefly luciferase reporter lacking miRNA-binding sites serves as control. Defects in miRISC assembly/function are expected to be reflected as an increase (de-repression) in reporter activity compared to the negative control. It is worth noting that employing a synthetic mature miRNA duplex in the assay effectively circumvents the confounding factor that SmD1 is required for miRNA biogenesis. In addition, we chose *let-7* because of its extremely low basal expression in S2 cells, thereby reducing background. Our analysis revealed that compared to control knockdown cells, SmD1-depleted cells display a marked de-repression of the *let-7* reporter, resembling the phenotype elicited by depletion of the core miRISC component AGO1 (Fig 4B). These data indicate that SmD1 is required for miRNA function. Consistent with this notion, we found that Flag-tagged AGO1, but not the control protein Ran, is capable of co-immunoprecipitating with endogenous SmD1 (Fig 4C). Furthermore, endogenous AGO1 as well as GW182, another component of the miRISC, were detected in SmD1 complex, but not in the control Ran complex or the control immunoprecipitates using a non-immune serum (Fig 4D and 4E). It appears that the SmD1-AGO1 and SmD1-GW182 interactions are not strongly affected by RNase treatment (Fig 4D and 4E). These data demonstrate the interaction between SmD1 and components of the miRISC.

Next, to determine the functional relevance of the SmD1-AGO1 interaction and to directly assess the role of SmD1 in miRISC assembly/function, we examined whether SmD1 depletion impairs the function of miRISC by measuring the slicer activity of AGO1-miRISC programmed by the *let-7* miRNA duplex. To circumvent the confounding factor that AGO2 is a much more robust slicer than AGO1 and thus could mask the weak slicer activity of AGO1, we first established stably transfected S2 cells expressing TAP-tagged AGO1. Then we immunopurified and immobilized the AGO1 complex onto agarose beads and incubated the AGO1 complex with cytoplasmic lysates (from either control cells or *SmD1* knockdown cells) together with the *let-7* miRNA duplex. The beads were subsequently washed thoroughly and the slicer activity of the bead-bound *let-7*-AGO1 miRISC against an mRNA substrate carrying a perfect *let-7* binding site was measured. This assay revealed a significantly weaker slicer activity of the AGO1-miRISC assembled in SmD1-depleted cell lysate than that assembled in control lysates (Fig 4F and 4G), suggesting that SmD1 is required for efficient miRISC assembly and/or function. To examine whether SmD1 is required for the loading of miRNAs into AGO1, which is the first step of miRISC assembly, we depleted SmD1 in TAP-AGO1-expressing S2 cells, and measured the levels of endogenous miRNAs in immuno-purified TAP-AGO1 complex (Fig 4H). Note that as a control for the amount of TAP-AGO1 expressed from the transgene across different samples, we used identical amount of cell lysates in each immunoprecipitation. Compared with controls, the absolute levels of both *miR-2b* and *miR-184* were markedly reduced in AGO1 miRISC recovered from SmD1-depleted cells (Fig 4H and 4I). This is in part due to lower levels of miRNAs in input samples from *SmD1* knockdown cells (Fig 4H, compare the input samples). In addition, we calculated AGO1 loading index by normalizing levels of AGO1-bound miRNAs against those in the input. This analysis revealed a moderate decrease in AGO1 loading index upon *SmD1* knockdown, suggesting that SmD1 is required for efficient loading of miRNAs into miRISC (Fig 4J). Taken together, these data demonstrate that SmD1 is required for efficient miRISC assembly/function besides its role in miRNA biogenesis.

### Transcriptome-wide identification of SmD1-associated RNAs

**Fig 5:**
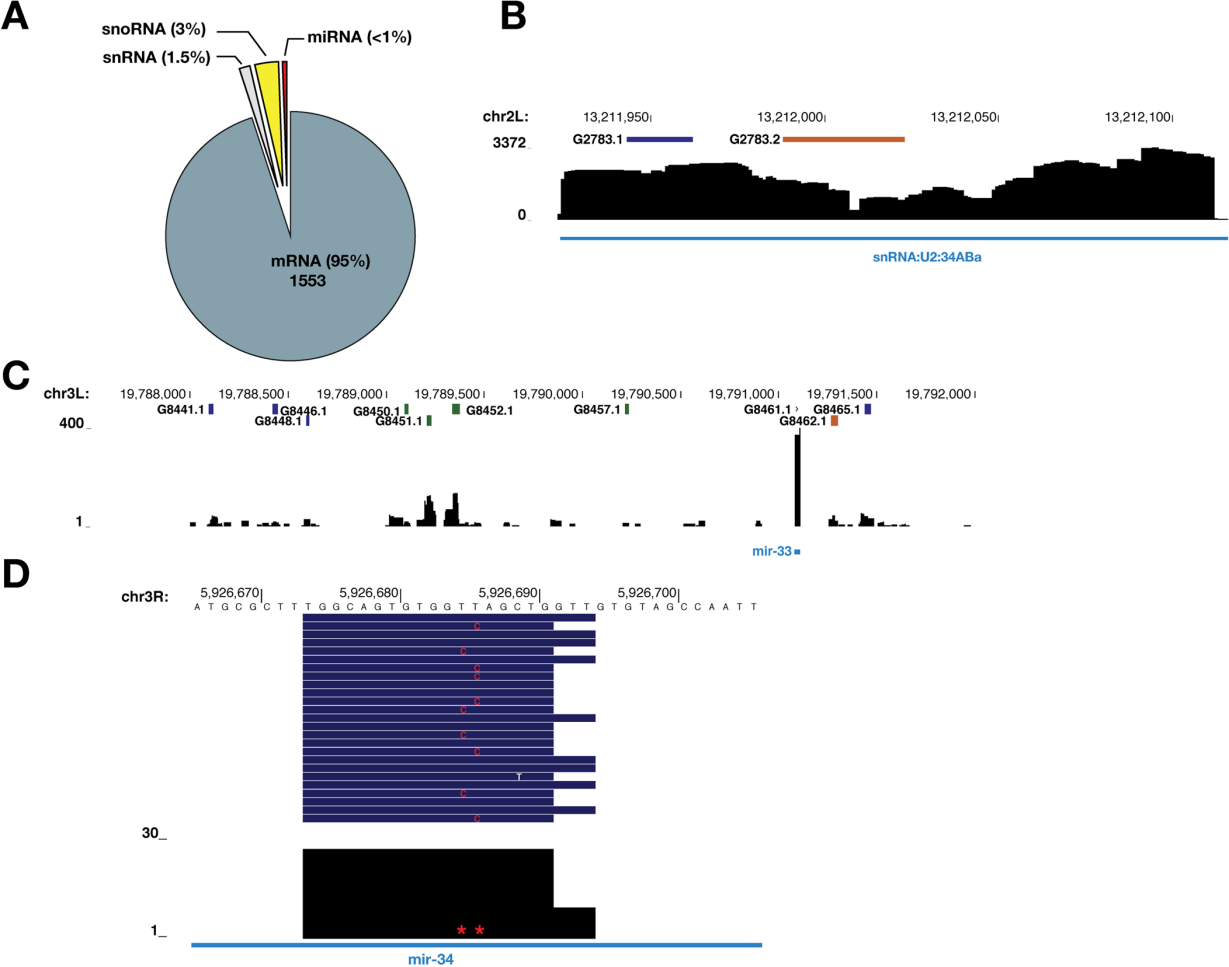
Transcriptome-wide identification of SmD1-associated RNAs. (**A**) A piechart showing read clusters derived from various classes of RNAs. Note that the rRNA sequences have been computationally removed (see Materials and Methods). (**B**) Binding signal in *snRNA*:*U2*:*34Aba* locus. Colored bars above the read coverage profile correspond to the number of T to C conversions identified in each cluster: Blue (2), green (3) and orange (≥ 4). Read depth is indicated on the left. (**C**) Binding profile in the *dme-miR-33* locus. Note that the G8461.1 binding site is of low confidence, due in part to a low proportion of T to C conversion events. (**D**) SmD1 binding site within mature *miR-34* sequence. T to C conversion loci are indicated with red asterisks. Reads mapping to this locus are shown as navy bars above the binding profile.

To comprehensively identify direct SmD1-RNA interaction events across the transcriptome, we optimized the PAR-CLIP protocol in *Drosophila* S2 cells, recovered and deeply sequenced SmD1-bound RNAs (S7A and S7B Fig) [41,42]. Mapped reads are derived from various classes of RNAs (S7C Fig). Our initial analysis identified 2180 SmD1 binding sites (clusters) across the transcriptome. Of these, 1729 unique peaks have passed non-adaptive filtration (see Materials and Methods) and were used for subsequent analyses (S2 Table). Among them, 96 map to unannotated genomic regions. For the remaining 1633 clusters, 1553 (95%) and 80 (5%), respectively, map to the annotated coding and non-coding RNAs (Figs 5A and S8; S3 and S4 Tables). As expected, snRNAs, the cognate binding partners for SmD1, were abundantly present in the dataset, so were sequences derived from the endogenous siRNA *esi-2*.*1* precursor *CG4068*, consistent with our previous report (Fig 5B and S2 Table) [33]. In addition, SmD1 binding events in the vicinity of splice junctions were found (S5 Table), thereby providing supporting evidence for well-documented role of SmD1 in regulation of pre-messenger RNA processing. Interestingly, we also identified a substantial number (24) of SmD1-interacting snoRNAs. Importantly, sequences derived from several pri-miRNAs, including *Bantam*, *miR-2* family, *miR-11*, *miR*-*33* and *miR*-*34* were also recovered, thus demonstrating direct interactions between SmD1 and pri-miRNAs (Figs 3C, 5C, 5D and S9; S6 Table). Interestingly, among the primary miRNA transcripts that we have examined so far, *miR-33* and *miR-34* are the top two highly enriched in immunopurified SmD1 complex (Figs 3C, 5C and 5D). Notably, a major SmD1 binding peak directly overlaps with mature *miR-34* (Fig 5D), suggesting that SmD1 may directly associate with mature miRNAs (in the context of miRISC). This is consistent with our previous findings that immunopurified SmD1 complex contains mature miRNAs [33]. An alternative and non-mutually exclusive possibility is that SmD1 could bind the sequence segment corresponding to mature *miR-34* within the primary miRNA transcript.

### Loss of SmD1 does not functionally alter the expression of canonical miRNA factors

**Fig 6:**
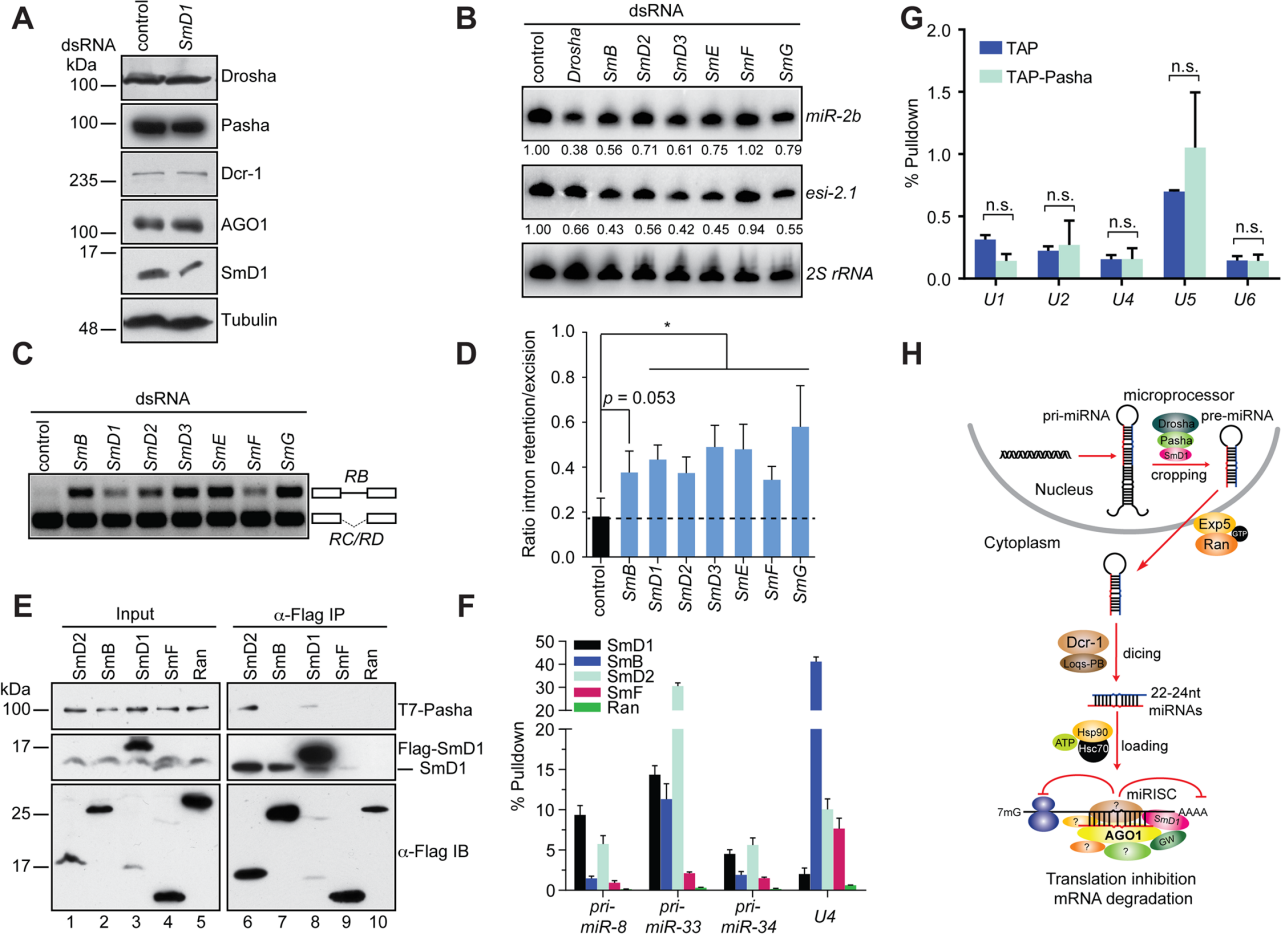
miRNA and splicing machineries are physically and functionally distinct. (**A**) S2 cells were treated with control or *SmD1* dsRNAs and analyzed for expression of various canonical miRNA pathway components by immunoblotting. Tubulin protein served as controls. Note identical copies of samples were subject to immunoblotting using various antibodies. (**B**) S2 cells were treated with various dsRNAs (top). Levels of *miR-2b*, *esi-2*.*1* and the control *2S* rRNA were measured by Northern blot and quantified (bottom). (**C,D**) Splicing activity was assessed in various knockdown cells (top) by examining splicing pattern of the *CG13887* pre-mRNA by RT-PCR. The RB splice variant retains an intron (a straight line between exons shown as open boxes), whereas the RC/RD variants lack the intron. Ratio of intron retention/excision was quantified and shown in **D** (n = 3). (**E**) Lysates from cells expressing various Flag-tagged Sm proteins or the control protein Ran together with T7-tagged Pasha were subject to immunoprecipitation using anti-Flag antibody. Both input samples and isolated complexes were probed with anti-T7 (top panel), anti-SmD1 (middle) and anti-Flag antibodies (bottom). (**F**) Lysates from cells expressing various Flag-tagged Sm proteins or the control protein were subject to immunoprecipitation using anti-Flag antibody. Total RNA was extracted from various immunopurified Flag-tagged protein complexes and subject to RT-qPCR to measure levels of various pri-miRNAs and the *U4* snRNA. (**G**) Total RNA was extracted from immunopurified TAP-Pasha or control samples (TAP) and subject to RT-qPCR to measure levels of various snRNAs. Fold change relative to levels of the control *rp49* mRNA are shown (n = 3; n.s., non-significant). (**H**) A schematic depicting the proposed roles of SmD1 in the initiation (miRNA biogenesis) and effector (miRISC assembly and/or mRNA cleavage) steps of the miRNA pathway.

It is conceivable that SmD1 depletion indirectly impacts the miRNA pathway by modulating the expression of genes encoding canonical miRNA factors. We previously performed pair-end RNA-sequencing in both control cells and cells depleted of SmD1 [33]. Consistent with the role of SmD1 in pre-mRNA splicing, our analysis revealed that the splicing pattern of ~25% cellular mRNAs is altered in SmD1-depleted cells. Importantly, we found no significant changes in the mRNA levels of canonical miRNA factors except an increase in levels of *Drosha* and a decrease in *loqs-RD*, whose encoded product is dispensable for the miRNA pathway (S10 Fig). Most importantly, immunoblotting assays revealed no significant changes in protein levels of canonical miRNA factors in SmD1-depleted cells except for an increase in levels of Loqs-PB and a concomitant reduction in Loqs-PD (Fig 6A) [33]. Given our findings that SmD1 impacts the cropping and miRISC assembly/function (Figs 2E–2G, 4 and S5), which do not require Loqs proteins, it is unlikely that the observed changes in levels of Loqs protein isoforms could account for the miRNA biogenesis and function defects elicited by SmD1 depletion. We conclude that SmD1 depletion does not functionally impact the expression of genes encoding canonical components of the miRNA pathway.

### Select splicing factors, but not splicing *per se*, modulate the miRNA pathway

To determine if a broader link exists between the miRNA pathway and splicing, we examined additional snRNP (Sm) proteins for their potential involvement in the miRNA pathway. We found that depletion of several snRNP proteins led a reduction in levels of *miR-2b*, reminiscent of the *SmD1* knockdown phenotype (Fig 6B). In contrast, levels of *miR-2b* remained unchanged in *SmF* knockdown cells. Furthermore, *SmF* knockdown did not impact in microprocessor activity either, even though SmF-depleted cells displayed obvious alteration in the splicing pattern of the *CG13887* pre-mRNA, to the same extent as that observed in SmD1-depleted cells (Figs S11, 6C and 6D). These data clearly show that the miRNA defects elicited by SmD1 depletion can be uncoupled from pre-mRNA splicing defects, as the miRNA pathway appears to be intact in SmF-depleted cells, even though these cells display profound defects in pre-mRNA splicing.

To further dissect the relationship between the miRNA and pre-mRNA splicing machineries, we examined whether additional snRNP proteins are capable of interacting with Pasha. We found that besides SmD1, SmD2 is also capable of co-immunoprecipitating with Pasha (Fig 6E). In contrast, no interaction can be detected between Pasha and SmB or SmF, even though both SmB and SmF are capable of pulling down endogenous SmD1, most likely the SmD1 fraction in the spliceosome (Fig 6E). Furthermore, upon examining levels of pri-miRNA and snRNA species in various immuno-purified Sm protein complexes, we found that SmD1 consistently outperforms SmF in binding to various pri-miRNAs. In contrast, we detected significantly higher levels of the *U4* snRNA in the SmF complex compared to those present in the SmD1 complex (Fig 6F). We note that Flag-tagged Sm proteins were employed in these assays and that these exogenous proteins have to complete with their endogenous counterparts for binding to their RNA partners. However, since we are measuring levels of various RNA cargos in the same sample, it allows us to make a direct comparison regarding the relative affinity between various Sm proteins and their RNA partners. These data suggest that compared with SmF, SmD1 appears to display a more prominent role in miRNA biogenesis, whereas SmF seems to be more dedicated to pre-mRNA splicing. In addition, these data also indicate that the spliceosome and miRISC are functionally distinct entities. To address this further, we immunopurified the microprocessor or the snRNP complexes by TAP-Pasha IP or SmD1 IP, respectively, and examined the presence of various snRNAs and pri-miRNAs in these complexes. As expected, snRNAs were highly enriched in the SmD1 complex (Fig 3C). In contrast, snRNAs were largely absent in the microprocessor (Fig 6G). On the other hand, several primary miRNA transcripts were highly enriched in the microprocessor (Figs 3D and S6). Taken together, these data demonstrate that select splicing factors, but not splicing *per se*, influence the miRNA pathway, and that the miRNA biogenesis machinery and the spliceosome are physically and functionally distinct.

## Discussion

Accumulating evidence suggests extensive crosstalk between pre-mRNA splicing and small RNA-mediated gene silencing pathways. For example, the core component of the RNAi effector machinery AGO2 plays a key role in the regulation of pre-mRNA splicing [19,20]. Conversely, a number of splicing factors have been implicated in modulating small RNA-mediated gene silencing [21,22,23,24,25,26,27,28,29,30]. We previously showed that the core snRNP splicing factor SmD1 is required for optimal biogenesis and function of siRNAs [33]. In the current study we report that SmD1 also plays a key role in modulating the miRNA pathway. Specifically, SmD1 interacts with the microprocessor component Pasha and with primary miRNA transcripts, and is selectively required for efficient recognition of primary miRNA transcripts by the microprocessor and the conversion of pri-miRNAs into pre-miRNAs. In addition, SmD1 associates with the miRISC components AGO1 and GW182, and is required for miRISC assembly/function. We further show that only select splicing factors, but not pre-mRNA splicing per se, modulate the miRNA pathway, since defects in pre-mRNA splicing can be uncoupled from defects in the miRNA pathway, and that the molecular machineries executing pre-mRNA splicing and miRNA biogenesis and function are physically and functionally distinct entities.

Our analysis reveals that SmD1, but not a closely related snRNP protein SmF, is required for optimal miRNA biogenesis. These observations indicate that SmD1 belongs to a select group of splicing factors that modulate small RNA pathways beyond the context of the splicing machinery. SmD1 associates with the N-terminus of Pasha, whereas the C-terminal domain of Pasha is sufficient to interact with Drosha. It is currently unclear whether the SmD1-Pasha and Drosha-Pasha interactions take place in a mutually exclusive manner, or alternatively there exists a Drosha-Pasha-SmD1 complex. Our observation that the immunopurified SmD1 complex is capable of carrying out the conversion of pri-miRNAs into pre-miRNAs lends support to the latter possibility (Fig 3B). Of note, several splicing factors have been shown to co-purify with the microprocessor [3]. Our study, together with a recent report that implicates FUS in miRNA biogenesis [43], demonstrates that select splicing factors are functionally associated with the microprocessor in higher multicellular organisms. While it is possible that SmD1 couples splicing and processing of pri-miRNA transcripts by recruiting the microprocessor to nascent pri-miRNA transcripts that undergo co-transcriptional splicing, our observations that depletion of SmF, a related snRNP splicing factor, does not impact miRNA biogenesis (Figs 6B and S11), that neither SmB nor SmF is able to co-immunoprecipitate with Pasha (Fig 6E), and that immunopurified SmD1 and Pasha complexes harbor overlapping yet distinct sets of cargo RNAs (Figs 3C, 6F and 6G), argue against this possibility.

While SmD1 is clearly required for miRNA biogenesis, it may operate in the context of multimeric protein complexes to execute this function. For example, our data show that SmD2 is as competent as SmD1 in pulling down primary miRNA transcripts and Pasha, and depletion of SmD2 led to a reduction in both miRNA levels and microprocessor activity (Figs 6B, 6E, 6F and S11). Together with previous studies that report the presence stable SmD1-SmD2 subcomplex, [31,44], our data raise the possibility that the SmD1/D2 sub-assembly may execute a moonlighting function during miRNA biogenesis beyond the context of the spliceosome. We found that adding back purified recombinant SmD1 or lysates from cells over-expressing SmD1 was not sufficient to restore the microprocessor activity or miRISC assembly/function in SmD1-depleted cell lysate (S12 and S13 Figs). These observations suggest that additional SmD1 co-factor(s) (such as SmD2) in the context of multimeric complexes of appropriate stoichiometry may be necessary to functionally impact the miRNA pathway. An alternative possibility is that SmD1 depletion leads to altered expression of unknown protein(s) involved in miRNA biology, which underlies the defects in the miRNA pathway elicited by *SmD1* knockdown. However, our findings lend strong support to the former scenario.

Interestingly, our data uncover a differential dedication of various Sm proteins to the miRNA pathway and pre-mRNA splicing. For example, SmD1 and SmD2 consistently outperform SmF in binding to various pri-miRNAs, whereas the *U4* snRNA is significantly more enriched in the SmF complex than in the SmD1 complex. (Fig 6F) This is consistent with our findings showing that SmD1 and SmD2, but not SmF, is required for the miRNA pathway. These observations also indicate the presence of sub-spliceosomal assemblies or novel SmD1-containing complexes that impact the miRNA pathway. Further supporting this notion, our co-immunoprecipitation assay reveals that Flag-tagged SmD1 is capable of pulling down endogenous SmD1 (Fig 6E, lane 8). Most likely this interaction takes place beyond the context of the spliceosome, as the snRNP ring structure contains only a single copy of each Sm protein. Interestingly, depleting either SmB or SmD2, immediate neighbors to SmD1 in the snRNP ring structure, led to a reduction in miRNA levels and microprocessor activity. However, only SmD2, but not SmB, is capable of co-immunoprecipitating with Pasha (Fig 6E). Identification of the complete collection of microprocessor-associated splicing factors and unraveling the stoichiometry of the microprocessor/SmD1-containing complexes should provide insights into the function of microprocessor-associated splicing factors in miRNA biogenesis.

Besides its involvement in the initiation phase of the miRNA pathway (miRNA biogenesis), SmD1 is also required for the effector phase of the miRNA pathway, as SmD1 depleted cells display defective miRISC assembly/function. This is manifested in part by defects in the loading of miRNAs into AGO1-miRISC (Fig 4H–4J). It remains to be determined whether SmD1 is similarly required for additional steps of miRISC assembly, including miRNA duplex unwinding and miRNA star strand removal.

We report here that the *Drosophila* SmD1 associates with the microprocessor and impacts the cropping step of miRNA biogenesis (Fig 6H). Interestingly, in *C*. *elegans* the SmD1 ortholog SNR-3 co-purifies with Dcr-1 [45]. It would be informative to address whether SNR-3 similarly impacts miRNA biogenesis in worms. In addition, we show here that SmD1 associates with the core miRISC component AGO1 in flies and impacts miRNA function (Fig 6H). An analogous observation has been made regarding the role of SmD1 in the siRNA pathway, where it associates with the siRISC component AGO2 and is required for siRISC assembly/function. Furthermore, we report previously that the human orthologs of SmD1 and AGO2 associate with each other [33]. These findings raise the possibility that the role of SmD1 in modulating small regulatory RNA biogenesis and Argonaute-RISC assembly and function may be evolutionarily conserved.

## Materials and Methods

### Cell culture, transfection, and RNAi

*Drosophila* S2 and S2-NP cells were maintained in Schneider’s medium (Invitrogen) supplemented with 10% fetal bovine serum (FBS) and 1% penicillin-streptomycin (Invitrogen). S2-NP cells stably expressing Flag-SmD1 were generated by transfection with pRmHa-3-Flag-SmD1 and the selection marker plasmid pHS-neo using the calcium phosphate method, followed by selection in medium containing 400 μg/mL G418 (Calbiochem). dsRNA treatment was performed as described previously [27,46]. Briefly, ~2 × 10^6^ S2 or S2-NP cells were seeded in 6-well plates for 24 h and then transfected with 3 μg of the appropriate dsRNA. Two days later, the cells were harvested, replated in 6-cm plates for 24 h, and then treated again with 9 μg dsRNA. Three days later, the cells were harvested and used in assays. For *Renilla* luciferase reporter assays, transfections were performed in a 384-well format using HiPerFect (Qiagen).

### DNA constructs and antibodies

DNA fragments encompassing the coding regions of SmD1, SmB, SmD2, SmF, Drosha, AGO1, and full length and truncated Pasha, together with Flag, T7 or TAP epitope tags were amplified by PCR and cloned into pRmHa-3 or pMK33-NTAP vectors. Anti-SmD1 antibody was generated by immunizing rabbits with a synthetic peptide (ProSci Inc.).

### Protein/RNA immunoprecipitation and immunoblotting

Cells were lysed in lysis buffer (20 mM Tris-HCl (pH 7.6), 150 mM NaCl, 2 mM EDTA, 10% glycerol, 1% Triton X-100, 1 mM DTT, 1 mM orthovanadate) supplemented with protease inhibitor cocktail (Roche). Cleared total lysates were immunoprecipitated with antibodies against Flag (Sigma). Both input and immunoprecipitated samples were analyzed by SDS-PAGE followed by immunoblotting with antibodies against Flag (Sigma), T7 (Novagen), AGO1 (Abcam), Pasha [5] or GW182 [47]. Other antibodies employed in this study include Drosha and Dcr-1 (gift from Dr. Greg Hannon), Loqs [48] or Tubulin (Sigma), as indicated. RNase treatment of the immunoprecipitates was performed as previously described [27,46]. To detect protein-RNA interactions, RNA was extracted from the immunoprecipitates by treatment with TRIzol and subject to RT-qPCR analysis using gene-specific primers.

### Northern blotting

Northern blotting was performed as previously described [46,48]. In brief, total cellular or co-immunoprecipitated RNA was isolated with TRIzol (Invitrogen). Samples of 15 μg RNA were separated on 15% denaturing polyacrylamide gels and transferred to Hybond-N+ membranes (Amersham Biosciences) in 1X TBE buffer. Small RNAs were UV crosslinked to the membranes, and the membranes were prehybridized in hybridization buffer for 2 h. DNA probes complementary to the appropriate strands were 5′ radiolabeled and incubated with membranes overnight at 37°C. Membranes were washed twice in 1X SSC with 0.1% SDS at 42°C, and then exposed to Phosphorimager screens for 12–48 h. Membranes were stripped by the addition of boiling 0.1% SDS solution and incubated for 30 min.

### Cropping, dicing and slicing assays

Cropping, dicing and slicing assays were performed as previously described, with minor modifications [49,50]. Briefly, for the cropping assay, total S2-NP cell lysates were prepared by sonicating cell suspension in lysis buffer (30 mM HEPES-KOH, pH 7.0, 100 mM potassium acetate, 2 mM magnesium acetate, 5 mM DTT, 20% glycerol, and 1X EDTA-free protease inhibitors (Roche)) for 5 times (5 sec each with 2 min interval at 30% duty cycle). Primary miRNAs were synthesized using a T7 MEGAscript *in vitro* transcription kit (Ambion) with α-^32^P-GTP, and gel purified. Aliquots of 10 μl of cell lysates containing the same amount of total protein were incubated in a final volume of 20 μl reaction mixture (30 mM HEPES-KOH, pH 7.0, 100 mM potassium acetate, 2 mM magnesium acetate, 5 mM DTT, 10% glycerol, 1 mM ATP, 10 mM creatine phosphate, 0.06 U/μl creatine kinase (Roche), 0.1 U/μl ribonuclease inhibitor (Promega), 10 ng/μl yeast tRNA, and 2000–10,000 cpm pri-miRNA substrate) at 25°C for 2.5 h.

For the dicing assay, cytoplasmic extracts from frozen S2-NP cells were prepared by thawing cells in a hypotonic buffer composed of 10 mM HEPES-KOH (pH 7.0), 2 mM magnesium acetate, 0.1% β-mercaptoethanol, and 1X EDTA-free protease inhibitors (Roche). Radiolabeled pre-miRNA substrate was prepared by incubating the *pre-let-7* synthetic RNA oligo with T4 polynucleotide kinase (New England Biolabs) and γ-^32^P-ATP and subsequently gel-purified. Aliquots of 6 μl of cell lysates containing the same amount of total protein were incubated in a final volume of 10 μl reaction mixture (20 mM HEPES-KOH, pH 7.0, 2 mM DTT, 2 mM magnesium chloride, 1 mM ATP, 25 mM creatine phosphate, 0.06 U/μl creatine kinase (Roche), 0.8 U/μl ribonuclease inhibitor (Promega), and 2000–10,000 cpm *pre-let-7* substrate) at 25°C for 1 h.

For the slicing assay, the capped *Renilla* luciferase mRNA substrate containing 1 copy of perfect *let-7* binding site in the 3’ UTR was synthesized using a MEGAscript T7 *in vitro* transcription kit, incubated with Vaccinia virus capping enzyme (New England Biolabs) and α-^32^P-GTP, and gel purified. Minimal AGO1-miRISC was prepared by incubating TAP-AGO1 cell lysates with IgG beads with gentle rocking at 4°C overnight. The beads were thoroughly washed in hypotonic buffer and incubated with cytoplasmic lysates from either *SmD1* knockdown or control cells in a final volume of 50 μl reaction mixture (8 mM HEPES-KOH (pH 7.0), 60 mM potassium acetate, 5 mM DTT, 1 mM ATP, 25 mM creatine phosphate, 0.03 U/μl creatine kinase, 0.2 U/μl ribonuclease inhibitor, and 1 mM *let-7* miRNA) at 25°C for 30 min. The beads were subsequently thoroughly washed in hypotonic buffer and incubated in a final volume of 50 μl reaction mixture (8 mM HEPES-KOH (pH 7.0), 60 mM potassium acetate, 5 mM DTT, 1 mM ATP, 25 mM creatine phosphate, 0.03 U/μl creatine kinase, 0.2 U/μl ribonuclease inhibitor, 10 ng/μl yeast tRNA, and 2000–10,000 cpm cap-labeled mRNA substrate) at 25°C for 2 h. After the final incubation step, the cropping, dicing or slicing reaction mixtures were then added to 200 μl proteinase K buffer (200 mM Tris-HCl (pH 7.5), 25 mM EDTA, 300 mM sodium chloride, 2% w/v SDS, and 50 μg/mL proteinase K), incubated at 65°C for 30 min, and extracted with phenol/chloroform (1:1). RNA was precipitated from the supernatant and resolved by 6% (for cropping and slicing) or 15% urea-PAGE (for dicing).

### PAR-CLIP and library construction and RNA-Seq data analysis

PAR-CLIP procedure and library construction were conducted as previously reported [41,42]. Sequenced reads were trimmed of 3’ and 5’ adaptors (3’ = "TGGAATTCTCGGGTGCCAAGG"; 5’ = "ATCTCGTATGCCGTCTTCTGCTTG") using flexbar package [51]. Reads that were less than 15 nucleotides in length were discarded. In order to accurately analyze each binding event without the confounding bias introduced by repetitive regions, we identified and removed reads that align to rRNA, tRNA and RepeatMasker sequences (UCSC BDGP R5/dm3 and FlyBase FB2014_03). Elimination of reads mapped to repetitive sequences has significantly affected the overall read yield available for further analyses, thus complicating discovery of SmD1 binding sites. The remaining reads were aligned to *Drosophila* genome with bowtie algorithm [52]. Mapped locations were only reported for the optimal mismatch-stratum for each read up to a maximum of ten different locations. The resulting alignment was processed with PARALYZER tool (v1.1) [53]. All clusters that have two or more T to C conversion locations were reported. The discovered clusters were further filtered to exclude those where a) read coverage was lower than 10, b) ModeScore< = 0.6, and c) number of unique locations having at least one conversion event exceeded 2. The above non-adaptive filtration helped to remove potential false positive binding events in low coverage regions, where PARALYZER’s signal-to-noise estimation becomes less reliable. The location that a cluster mapped to, relative to a known transcript, was determined based on the FlyBase genome annotation (release 5.57). To assess the extent of SmD1 binding in miRNA precursor regions, ±10Kb around the known miRNA locus were examined and overlapping read clusters detected with BEDTools suite. To evaluate the role of SmD1 on regulation of mRNA splicing, coordinates of exon junctions annotated in FlyBase were extracted. Next, regions of interest around exon–intron junctions at the 5′ and 3′ ends of introns were determined. These loci included: a) 15 bp region around 5’ splice site, of which 5bp were inside the intron, and b) 28bp region around 3’ splice site, where only 3bp overlapped with the 3’ exon. Finally, the intersection between SmD1 binding and mRNA splice sites was computed.

### Small RNA library construction and data analysis

Small RNA libraries were constructed from gel purified 19–24 nt RNA samples using the TruSeq small RNA sample kit according to manufacturer’s manual (Illumina), and sequenced on a GA-II machine. Reads were trimmed of 3’ and 5’ adaptors (3’ = "TGGAATTCTCGGGTGCCAAGG"; 5’ = "ATCTCGTATGCCGTCTTCTGCTTG") with cutadapt package (http://dx.doi.org/10.14806/ej.17.1.200) and mapped to the fly genome (rel. 5.57) with Bowtie software suite (v.1.1.1) [52] in a sequential manner {e.g. progressively relaxing constraints for permissive number of mismatches until the limit of 2 was reached (–v[0–2]-best)}. The alignment was processed with HTSeq [54] and read abundances assigned to various genomic elements calculated. To assess read numbers mapping to transposable elements loci we used only LINE-like and LTR elements annotated in RepeatMasker (UCSC BDGP R5/dm3 and FlyBase FB2014_03). Annotation and genomic coordinates for 3p-CIS-NAT, esiRNA loci used to estimate siRNA abundances were obtained from Eric Lai (personal communication). Reads that failed to map to the fly genome were aligned to the flock house virus (FHV) genome and counted.

#### Data availability

The sequencing results will be submitted to the SRA/ENA repository.

### Oligonucleotides

See S7 Table.

### Adult fly heart analysis

*SmD1* was knocked down in the *Drosophila* heart by cardiac-specific RNAi using the UAS/Gal4 system [55]. Female *tinC*Δ*4*-Gal4 flies [56] were crossed to UAS-*shSmD1* (Bloomington stock 34834) and UAS-*shGFP* (gift from Dr. Norbert Perrimon) respectively. The female F1 progeny was aged for 3 weeks at 25°C and heart function was analyzed using high-speed recordings of semi-dissected hearts [57].

## Supporting Information

S1 Fig Two independent dsRNAs against *SmD1* cause on-target phenotypes. (**A**) S2 cells were treated with various dsRNAs (above) and levels of *esi-2*.*1*, various miRNAs or *2S* rRNA (loading control) were measured by Northern blot. Two independent dsRNAs targeting *SmD1* (*DRSC09800* and *DRSC31709*) cause a similar phenotype. (**B**) Quantification of miRNA and *esi-2*.*1* levels (n = 2) normalized against *2S* rRNA levels and compared with controls.
(TIF)

S2 Fig Loss of SmD1 led to a reduction in *let-7* levels. Various genes (labeled on the top) were inactivated by dsRNAs in S2 cells. Cells were then treated with 20-hydroxyecdysone (**20E**) for 48 h prior to harvest. Total RNAs were isolated and levels of *let-7* and *esi-2*.*1* were analyzed by Northern blot. Quantifications of *let-7* and *esi-2*.*1* levels normalized against *2S* rRNA levels are shown at the bottom.
(TIF)

S3 Fig *SmD1* inactivation impairs cardiac function. Bar graph representations of (**A**) systolic interval lengths, (**B**) diastolic diameters and (**C**) fractional shortening in *SmD1* RNAi hearts (3 week old females). The cardiac-specific *tinCΔ4-Gal4* driver and UAS-shRNA lines were employed to deplete SmD1 in the fly heart. *SmD1* inactivation caused a decrease in the three parameters analyzed compared to the age-matched control flies (*sh-GFP*). Results are the mean + SD (*sh-GFP*, n = 14; *sh-SmD1*, n = 18; * p < 0.05; ** p < 0.01, one-way ANOVA).
(TIF)

S4 Fig Drosha depletion causes accumulation of pri-miRNAs. Levels of various primary miRNA transcripts in Drosha-depleted cells or control cells were measured by RT-qPCR and normalized against the control *rp49* mRNA (n ≥ 3).
(TIF)

S5 Fig Depletion of SmD1 impairs microprocessor activity. Microprocessor activities in lysates from various knockdown cells (top) were assayed using four pri-miRNAs as substrates. Quantifications of microprocessor activity are shown at the bottom.
(TIF)

S6 Fig Primary miRNA transcripts are enriched in immuno-purified Pasha complexes. Total RNA was extracted from immunopurified TAP-Pasha or control samples (TAP) and subject to RT-qPCR to measure levels of various pri-miRNAs. Percentage of enrichment relative to the input samples are shown (n = 4; **p < 0.01).
(TIF)

S7 Fig PAR-CLIP identifies SmD1-associated RNAs. **(A)** S2 cells expressing TAP-SmD1 were cultured in the presence or absence of 4-thio-uridine (**4-SU**), and irradiated with 365 nm UV. Crosslinked protein-RNA complexes were immunopurified using IgG agarose, treated with RNase T1 to fragment RNAs, radiolabeled with T4 polynucleotide kinase, subject to SDS-PAGE, transferred to nitrocellulose membrane and visualized by phosphorimager. An immunoblot (**IB**) of the non-crosslinked TAP-SmD1 is shown on the right. (**B**) RNA was extracted from membrane slices (marked by a dashed rectangle in **A**), subject to 6% Urea-PAGE, and detected by autoradiography. (**C**) A piechart showing raw read counts and corresponding percentages of mapped reads derived from various classes of RNAs.
(TIF)

S8 Fig A circos plot showing the distribution of mapped SmD1 PAR-CLIP clusters across the genome. Scaled curves at the outer circle indicate various chromosomes, whereas clusters mapped to the coding and non-coding RNAs are illustrated with filled circles in red and black, respectively. The size of the filled circles reflects the modeScore value for every cluster [score of the highest signal / (signal + background)] value generated by PARalyzer. Select miRNAs are indicated inside the circle.
(TIF)

S9 Fig High magnification of *miR-33* precursor region. **A** and **B** illustrate two SmD1-bound clusters at the *miR-33* locus, corresponding read coverage and T to C conversion events are shown.
(TIF)

S10 Fig Loss of SmD1 does not significantly affect the expression of canonical miRNA pathway components. (**A**) RNA samples from *SmD1* knockdown cells or controls cells were subject to RNA sequencing. Read counts for various splice variants of canonical miRNA pathway components are shown. Upon normalization to the control *rp49* mRNA, fold changes in mRNA levels of canonical miRNA factors in SmD1-depleted cells relative to control samples are calculated. (**B**) RT-qPCR was employed to validate the deep sequencing results from **A**.
(TIF)

S11 Fig Loss of SmF does not impair microprocessor activity. (**A,B**) Microprocessor activities in lysates from various dsRNA-treated cells (top) were assayed in **A** and quantification results are shown in **B** (n ≥ 3; mean + SEM; **p < 0.01).
(TIF)

S12 Fig Recombinant SmD1 or lysates from *SmD1*-overexpression cells do not restore the microprocessor activity in lysates from *SmD1*-knockdown cells. Various combinations of recombinant SmD1 or lysates from *SmD1*-overexpression cells or *SmD1*-knockdown cells were subject to microprocessor assay using *pri-miR-34* as substrate.
(TIF)

S13 Fig Recombinant SmD1 or lysates from *SmD1*-overexpression cells do not restore the miRISC activity in lysates from *SmD1*-knockdown cells. Various combinations of recombinant SmD1 or lysates from *SmD1*-overexpression cells or *SmD1*-knockdown cells were subject to AGO1-miRISC slicing assay as described in **4F**.
(TIF)

S1 Table Read counts of various class of endogenous siRNAs and miRNAs in cells depleted of Drosha, Dcr-2 or SmD1. (XLSX)

S2 Table SmD1-binding sites across the *Drosophila* transcriptome. (XLSX)

S3 Table SmD1-binding sites that map to coding regions. (XLSX)

S4 Table SmD1-binding sites that map to non-coding regions. (XLSX)

S5 Table SmD1-binding sites that map across exon-intron junctions. (XLSX)

S6 Table SmD1-binding sites that map to pri-miRNAs. (XLSX)

S7 Table Oligos employed in this study. (XLSX)

## References

[1] Reinhart BJ, Slack FJ, Basson M, Pasquinelli AE, Bettinger JC, et al (2000) The 21-nucleotide let-7 RNA regulates developmental timing in Caenorhabditis elegans. Nature 403: 901–906. 10706289 10.1038/35002607

[2] Lee RC, Feinbaum RL, Ambros V (1993) The C. elegans heterochronic gene lin-4 encodes small RNAs with antisense complementarity to lin-14. Cell 75: 843–854. 8252621 10.1016/0092-8674(93)90529-y

[3] Gregory RI, Yan KP, Amuthan G, Chendrimada T, Doratotaj B, et al (2004) The Microprocessor complex mediates the genesis of microRNAs. Nature 432: 235–240. 15531877 10.1038/nature03120

[4] Lee Y, Ahn C, Han J, Choi H, Kim J, et al (2003) The nuclear RNase III Drosha initiates microRNA processing. Nature 425: 415–419. 14508493 10.1038/nature01957

[5] Denli AM, Tops BB, Plasterk RH, Ketting RF, Hannon GJ (2004) Processing of primary microRNAs by the Microprocessor complex. Nature 432: 231–235. 15531879 10.1038/nature03049

[6] Saito K, Ishizuka A, Siomi H, Siomi MC (2005) Processing of pre-microRNAs by the Dicer-1-Loquacious complex in Drosophila cells. PLoS Biol 3: e235 15918769 10.1371/journal.pbio.0030235 PMC1141268

[7] Hutvagner G, McLachlan J, Pasquinelli AE, Balint E, Tuschl T, et al (2001) A cellular function for the RNA-interference enzyme Dicer in the maturation of the let-7 small temporal RNA. Science 293: 834–838. 11452083 10.1126/science.1062961

[8] Forstemann K, Tomari Y, Du T, Vagin VV, Denli AM, et al (2005) Normal microRNA maturation and germ-line stem cell maintenance requires Loquacious, a double-stranded RNA-binding domain protein. PLoS Biol 3: e236 15918770 10.1371/journal.pbio.0030236 PMC1141267

[9] Jiang F, Ye X, Liu X, Fincher L, McKearin D, et al (2005) Dicer-1 and R3D1-L catalyze microRNA maturation in Drosophila. Genes Dev 19: 1674–1679. 15985611 10.1101/gad.1334005 PMC1176004

[10] Lund E, Guttinger S, Calado A, Dahlberg JE, Kutay U (2004) Nuclear export of microRNA precursors. Science 303: 95–98. 14631048 10.1126/science.1090599

[11] Yi R, Qin Y, Macara IG, Cullen BR (2003) Exportin-5 mediates the nuclear export of pre-microRNAs and short hairpin RNAs. Genes Dev 17: 3011–3016. 14681208 10.1101/gad.1158803 PMC305252

[12] Bohnsack MT, Czaplinski K, Gorlich D (2004) Exportin 5 is a RanGTP-dependent dsRNA-binding protein that mediates nuclear export of pre-miRNAs. RNA 10: 185–191. 14730017 10.1261/rna.5167604 PMC1370530

[13] Guo H, Ingolia NT, Weissman JS, Bartel DP (2010) Mammalian microRNAs predominantly act to decrease target mRNA levels. Nature 466: 835–840. 10.1038/nature09267 20703300 PMC2990499

[14] Pillai RS, Bhattacharyya SN, Artus CG, Zoller T, Cougot N, et al (2005) Inhibition of translational initiation by Let-7 MicroRNA in human cells. Science 309: 1573–1576. 16081698 10.1126/science.1115079

[15] Fabian MR, Mathonnet G, Sundermeier T, Mathys H, Zipprich JT, et al (2009) Mammalian miRNA RISC recruits CAF1 and PABP to affect PABP-dependent deadenylation. Mol Cell 35: 868–880. 10.1016/j.molcel.2009.08.004 19716330 PMC2803087

[16] Lewis BP, Shih IH, Jones-Rhoades MW, Bartel DP, Burge CB (2003) Prediction of mammalian microRNA targets. Cell 115: 787–798. 14697198 10.1016/s0092-8674(03)01018-3

[17] Lai EC, Tam B, Rubin GM (2005) Pervasive regulation of Drosophila Notch target genes by GY-box-, Brd-box-, and K-box-class microRNAs. Genes Dev 19: 1067–1080. 15833912 10.1101/gad.1291905 PMC1091741

[18] Lai EC, Burks C, Posakony JW (1998) The K box, a conserved 3' UTR sequence motif, negatively regulates accumulation of enhancer of split complex transcripts. Development 125: 4077–4088. 9735368 10.1242/dev.125.20.4077

[19] Taliaferro JM, Aspden JL, Bradley T, Marwha D, Blanchette M, et al (2013) Two new and distinct roles for Drosophila Argonaute-2 in the nucleus: alternative pre-mRNA splicing and transcriptional repression. Genes Dev 27: 378–389. 10.1101/gad.210708.112 23392611 PMC3589555

[20] Ameyar-Zazoua M, Rachez C, Souidi M, Robin P, Fritsch L, et al (2012) Argonaute proteins couple chromatin silencing to alternative splicing. Nat Struct Mol Biol 19: 998–1004. 10.1038/nsmb.2373 22961379

[21] Bayne EH, Portoso M, Kagansky A, Kos-Braun IC, Urano T, et al (2008) Splicing factors facilitate RNAi-directed silencing in fission yeast. Science 322: 602–606. 10.1126/science.1164029 18948543 PMC2585287

[22] Herr AJ, Molnar A, Jones A, Baulcombe DC (2006) Defective RNA processing enhances RNA silencing and influences flowering of Arabidopsis. Proc Natl Acad Sci U S A 103: 14994–15001. 17008405 10.1073/pnas.0606536103 PMC1581427

[23] Guil S, Caceres JF (2007) The multifunctional RNA-binding protein hnRNP A1 is required for processing of miR-18a. Nat Struct Mol Biol 14: 591–596. 17558416 10.1038/nsmb1250

[24] Michlewski G, Caceres JF (2010) Antagonistic role of hnRNP A1 and KSRP in the regulation of let-7a biogenesis. Nat Struct Mol Biol 17: 1011–1018. 10.1038/nsmb.1874 20639884 PMC2923024

[25] Ruggiero T, Trabucchi M, De Santa F, Zupo S, Harfe BD, et al (2009) LPS induces KH-type splicing regulatory protein-dependent processing of microRNA-155 precursors in macrophages. FASEB J 23: 2898–2908. 10.1096/fj.09-131342 19423639

[26] Trabucchi M, Briata P, Garcia-Mayoral M, Haase AD, Filipowicz W, et al (2009) The RNA-binding protein KSRP promotes the biogenesis of a subset of microRNAs. Nature 459: 1010–1014. 10.1038/nature08025 19458619 PMC2768332

[27] Zhou R, Hotta I, Denli AM, Hong P, Perrimon N, et al (2008) Comparative analysis of argonaute-dependent small RNA pathways in Drosophila. Mol Cell 32: 592–599. 10.1016/j.molcel.2008.10.018 19026789 PMC2615197

[28] Kim JK, Gabel HW, Kamath RS, Tewari M, Pasquinelli A, et al (2005) Functional genomic analysis of RNA interference in C. elegans. Science 308: 1164–1167. 15790806 10.1126/science.1109267

[29] Parry DH, Xu J, Ruvkun G (2007) A whole-genome RNAi Screen for C. elegans miRNA pathway genes. Curr Biol 17: 2013–2022. 18023351 10.1016/j.cub.2007.10.058 PMC2211719

[30] Tabach Y, Billi AC, Hayes GD, Newman MA, Zuk O, et al (2013) Identification of small RNA pathway genes using patterns of phylogenetic conservation and divergence. Nature 493: 694–698. 10.1038/nature11779 23364702 PMC3762460

[31] Kambach C, Walke S, Young R, Avis JM, de la Fortelle E, et al (1999) Crystal structures of two Sm protein complexes and their implications for the assembly of the spliceosomal snRNPs. Cell 96: 375–387. 10025403 10.1016/s0092-8674(00)80550-4

[32] Mount SM, Salz HK (2000) Pre-messenger RNA processing factors in the Drosophila genome. J Cell Biol 150: F37–44. 10908584 10.1083/jcb.150.2.f37 PMC2180228

[33] Xiong XP, Kurthkoti K, Chang KY, Lichinchi G, De N, et al (2013) Core small nuclear ribonucleoprotein particle splicing factor SmD1 modulates RNA interference in Drosophila. Proc Natl Acad Sci U S A 110: 16520–16525. 10.1073/pnas.1315803110 24067655 PMC3799365

[34] Brennecke J, Hipfner DR, Stark A, Russell RB, Cohen SM (2003) bantam encodes a developmentally regulated microRNA that controls cell proliferation and regulates the proapoptotic gene hid in Drosophila. Cell 113: 25–36. 12679032 10.1016/s0092-8674(03)00231-9

[35] Stark A, Brennecke J, Russell RB, Cohen SM (2003) Identification of Drosophila MicroRNA targets. PLoS Biol 1: E60 14691535 10.1371/journal.pbio.0000060 PMC270017

[36] Sempere LF, Sokol NS, Dubrovsky EB, Berger EM, Ambros V (2003) Temporal regulation of microRNA expression in Drosophila melanogaster mediated by hormonal signals and broad-Complex gene activity. Dev Biol 259: 9–18. 12812784 10.1016/s0012-1606(03)00208-2

[37] Kadener S, Rodriguez J, Abruzzi KC, Khodor YL, Sugino K, et al (2009) Genome-wide identification of targets of the drosha-pasha/DGCR8 complex. RNA 15: 537–545. 10.1261/rna.1319309 19223442 PMC2661833

[38] Han J, Pedersen JS, Kwon SC, Belair CD, Kim YK, et al (2009) Posttranscriptional crossregulation between Drosha and DGCR8. Cell 136: 75–84. 10.1016/j.cell.2008.10.053 19135890 PMC2680683

[39] Yeom KH, Lee Y, Han J, Suh MR, Kim VN (2006) Characterization of DGCR8/Pasha, the essential cofactor for Drosha in primary miRNA processing. Nucleic Acids Res 34: 4622–4629. 16963499 10.1093/nar/gkl458 PMC1636349

[40] Nguyen TA, Jo MH, Choi YG, Park J, Kwon SC, et al (2015) Functional Anatomy of the Human Microprocessor. Cell 161: 1374–1387. 10.1016/j.cell.2015.05.010 26027739

[41] Hafner M, Landthaler M, Burger L, Khorshid M, Hausser J, et al (2010) Transcriptome-wide identification of RNA-binding protein and microRNA target sites by PAR-CLIP. Cell 141: 129–141. 10.1016/j.cell.2010.03.009 20371350 PMC2861495

[42] Martin G, Gruber AR, Keller W, Zavolan M (2012) Genome-wide analysis of pre-mRNA 3' end processing reveals a decisive role of human cleavage factor I in the regulation of 3' UTR length. Cell Rep 1: 753–763. 10.1016/j.celrep.2012.05.003 22813749

[43] Morlando M, Dini Modigliani S, Torrelli G, Rosa A, Di Carlo V, et al (2012) FUS stimulates microRNA biogenesis by facilitating co-transcriptional Drosha recruitment. EMBO J 31: 4502–4510. 10.1038/emboj.2012.319 23232809 PMC3545295

[44] Lehmeier T, Raker V, Hermann H, Luhrmann R (1994) cDNA cloning of the Sm proteins D2 and D3 from human small nuclear ribonucleoproteins: evidence for a direct D1-D2 interaction. Proc Natl Acad Sci U S A 91: 12317–12321. 7527560 10.1073/pnas.91.25.12317 PMC45428

[45] Duchaine TF, Wohlschlegel JA, Kennedy S, Bei Y, Conte D Jr., et al (2006) Functional proteomics reveals the biochemical niche of C. elegans DCR-1 in multiple small-RNA-mediated pathways. Cell 124: 343–354. 16439208 10.1016/j.cell.2005.11.036

[46] Czech B, Malone CD, Zhou R, Stark A, Schlingeheyde C, et al (2008) An endogenous small interfering RNA pathway in Drosophila. Nature 453: 798–802. 10.1038/nature07007 18463631 PMC2895258

[47] Miyoshi K, Okada TN, Siomi H, Siomi MC (2009) Characterization of the miRNA-RISC loading complex and miRNA-RISC formed in the Drosophila miRNA pathway. RNA 15: 1282–1291. 10.1261/rna.1541209 19451544 PMC2704077

[48] Zhou R, Czech B, Brennecke J, Sachidanandam R, Wohlschlegel JA, et al (2009) Processing of Drosophila endo-siRNAs depends on a specific Loquacious isoform. RNA 15: 1886–1895. 10.1261/rna.1611309 19635780 PMC2743050

[49] Ishizuka A, Saito K, Siomi MC, Siomi H (2006) In vitro precursor microRNA processing assays using Drosophila Schneider-2 cell lysates. Methods Mol Biol 342: 277–286. 16957382 10.1385/1-59745-123-1:277

[50] Nayak A, Andino R (2011) Slicer activity in Drosophila melanogaster S2 extract. Methods Mol Biol 721: 231–244. 10.1007/978-1-61779-037-9_14 21431689

[51] Dodt M, Roehr JT, Ahmed R, Dieterich C (2012) FLEXBAR-Flexible Barcode and Adapter Processing for Next-Generation Sequencing Platforms. Biology (Basel) 1: 895–905. 24832523 10.3390/biology1030895 PMC4009805

[52] Langmead B, Trapnell C, Pop M, Salzberg SL (2009) Ultrafast and memory-efficient alignment of short DNA sequences to the human genome. Genome Biol 10: R25 10.1186/gb-2009-10-3-r25 19261174 PMC2690996

[53] Corcoran DL, Georgiev S, Mukherjee N, Gottwein E, Skalsky RL, et al (2011) PARalyzer: definition of RNA binding sites from PAR-CLIP short-read sequence data. Genome Biol 12: R79 10.1186/gb-2011-12-8-r79 21851591 PMC3302668

[54] Anders S, Pyl PT, Huber W (2015) HTSeq—a Python framework to work with high-throughput sequencing data. Bioinformatics 31: 166–169. 10.1093/bioinformatics/btu638 25260700 PMC4287950

[55] Brand AH, Perrimon N (1993) Targeted gene expression as a means of altering cell fates and generating dominant phenotypes. Development 118: 401–415. 8223268 10.1242/dev.118.2.401

[56] Lo PC, Skeath JB, Gajewski K, Schulz RA, Frasch M (2002) Homeotic genes autonomously specify the anteroposterior subdivision of the Drosophila dorsal vessel into aorta and heart. Dev Biol 251: 307–319. 12435360 10.1006/dbio.2002.0839

[57] Fink M, Callol-Massot C, Chu A, Ruiz-Lozano P, Izpisua Belmonte JC, et al (2009) A new method for detection and quantification of heartbeat parameters in Drosophila, zebrafish, and embryonic mouse hearts. Biotechniques 46: 101–113. 10.2144/000113078 19317655 PMC2855226

