# Supplementary figures and images for: SmD1 Modulates the miRNA Pathway Independently of Its Pre-mRNA Splicing Function
Source: PLoS Genet. 2015 Aug 26;11(8):e1005475. doi: 10.1371/journal.pgen.1005475 (PMC4550278; doi:10.1371/journal.pgen.1005475)

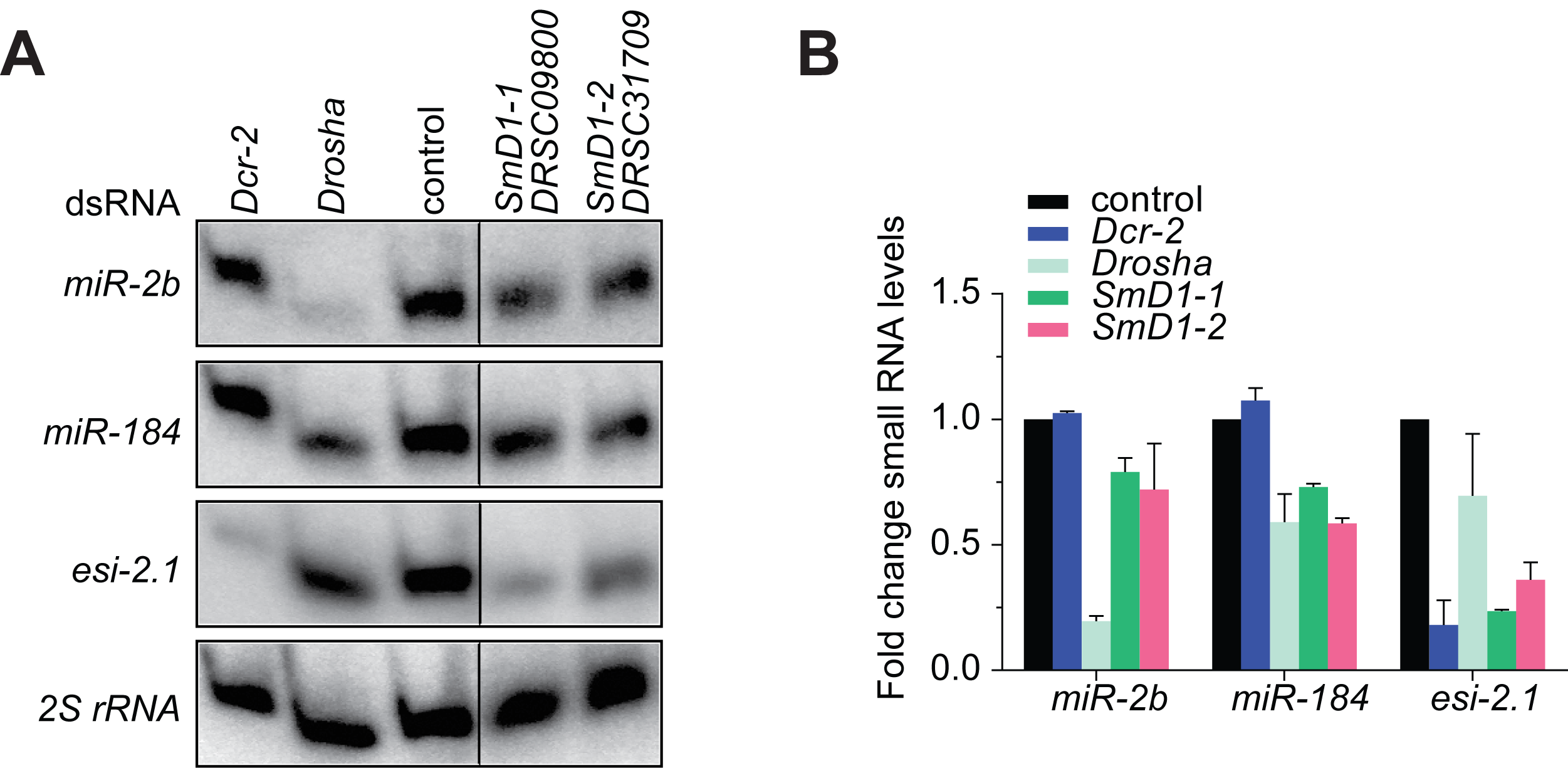

Supplement: S1 Fig — (A) S2 cells were treated with various dsRNAs (above) and levels of esi-2.1, various miRNAs or 2S rRNA (loading control) were measured by Northern blot. Two independent dsRNAs targeting SmD1 (DRSC09800 and DRSC31709) cause a similar phenotype. (B) Quantification of miRNA and esi-2.1 levels (n = 2) normalized against 2S rRNA levels and compared with controls. (TIF) [file pgen.1005475.s001.tif]

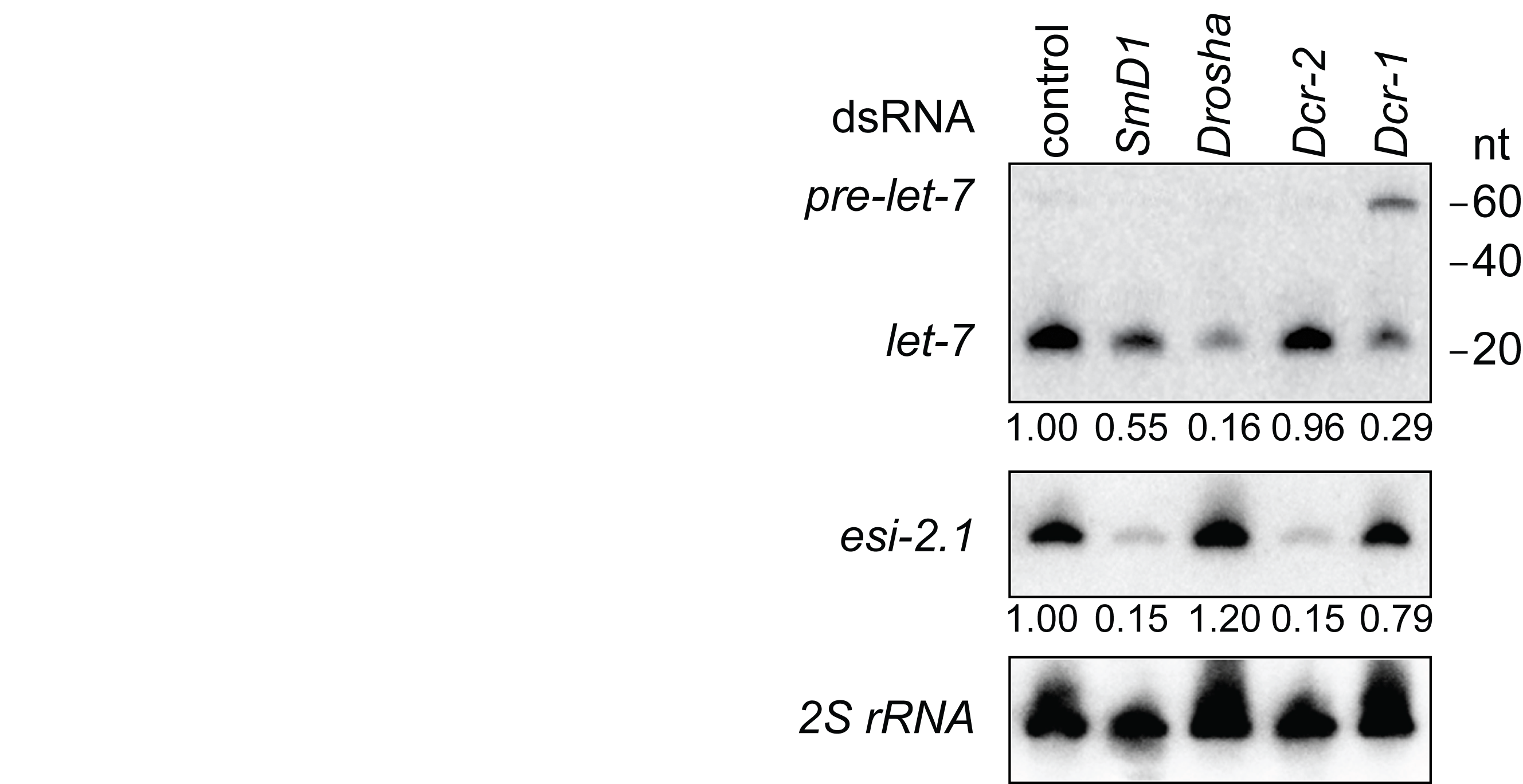

Supplement: S2 Fig — Various genes (labeled on the top) were inactivated by dsRNAs in S2 cells. Cells were then treated with 20-hydroxyecdysone (20E) for 48 h prior to harvest. Total RNAs were isolated and levels of let-7 and esi-2.1 were analyzed by Northern blot. Quantifications of let-7 and esi-2.1 levels normalized against 2S rRNA levels are shown at the bottom. (TIF) [file pgen.1005475.s002.tif]

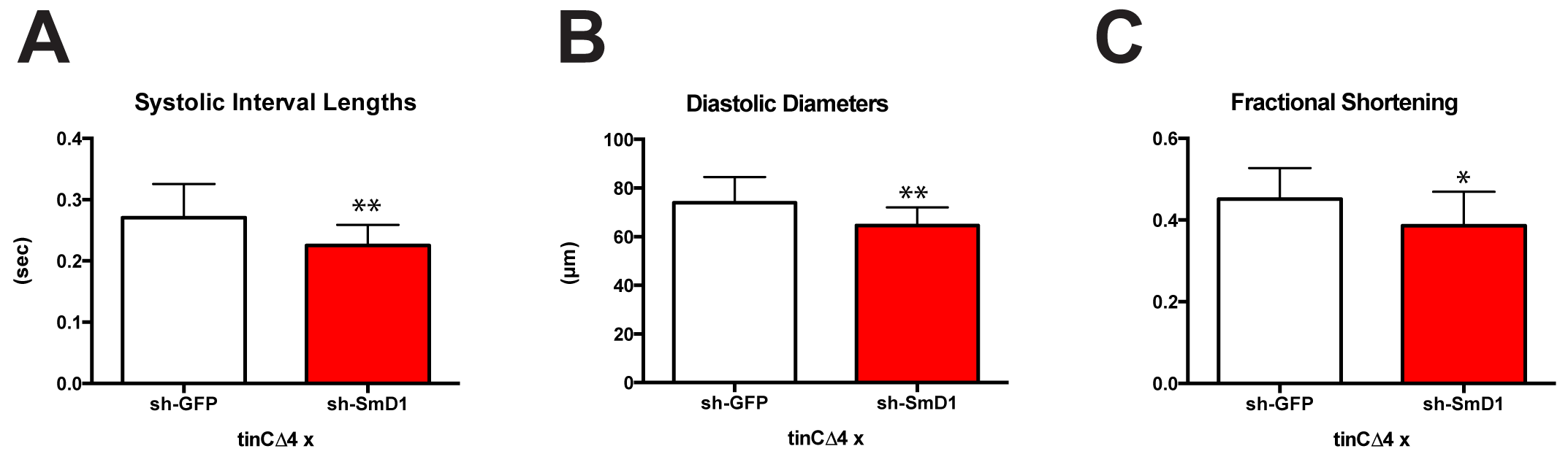

Supplement: S3 Fig — Bar graph representations of (A) systolic interval lengths, (B) diastolic diameters and (C) fractional shortening in SmD1 RNAi hearts (3 week old females). The cardiac-specific tinCΔ4-Gal4 driver and UAS-shRNA lines were employed to deplete SmD1 in the fly heart. SmD1 inactivation caused a decrease in the three parameters analyzed compared to the age-matched control flies (sh-GFP). Results are the mean + SD (sh-GFP, n = 14; sh-SmD1, n = 18; * p < 0.05; ** p < 0.01, one-way ANOVA). (TIF) [file pgen.1005475.s003.tif]

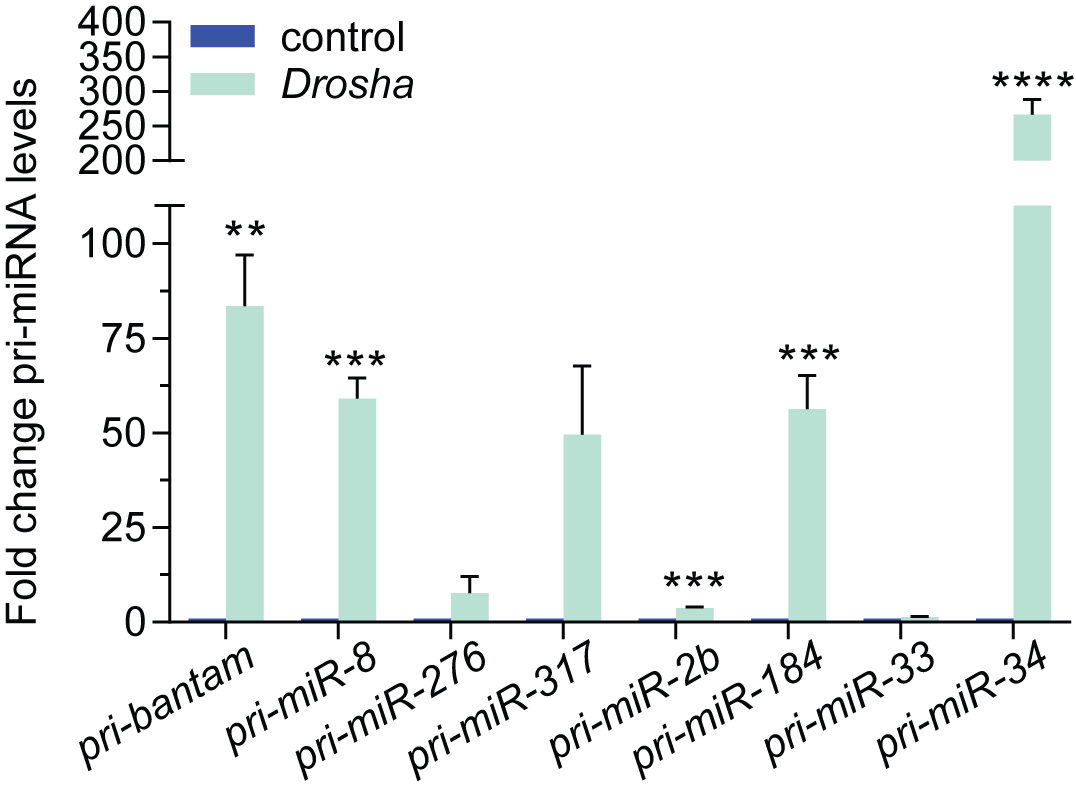

Supplement: S4 Fig — Levels of various primary miRNA transcripts in Drosha-depleted cells or control cells were measured by RT-qPCR and normalized against the control rp49 mRNA (n ≥ 3). (TIF) [file pgen.1005475.s004.tif]

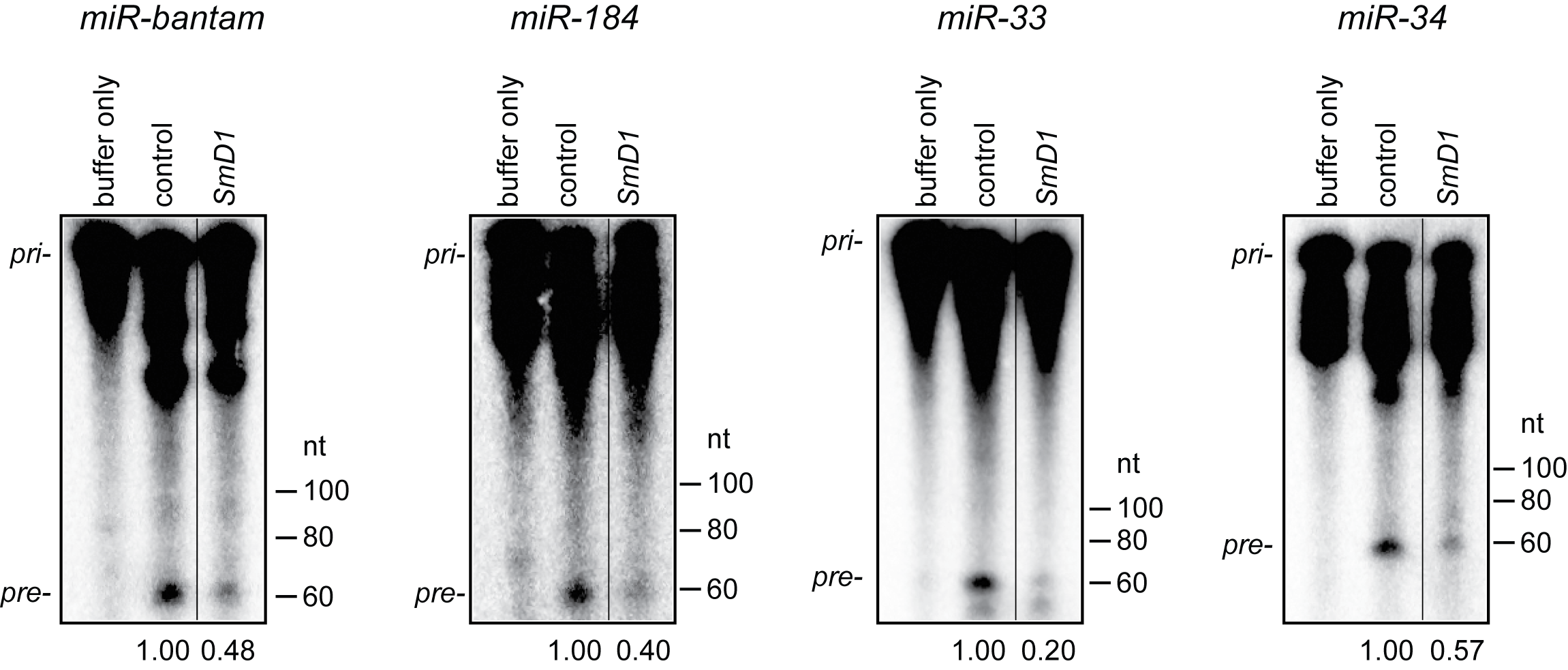

Supplement: S5 Fig — Microprocessor activities in lysates from various knockdown cells (top) were assayed using four pri-miRNAs as substrates. Quantifications of microprocessor activity are shown at the bottom. (TIF) [file pgen.1005475.s005.tif]

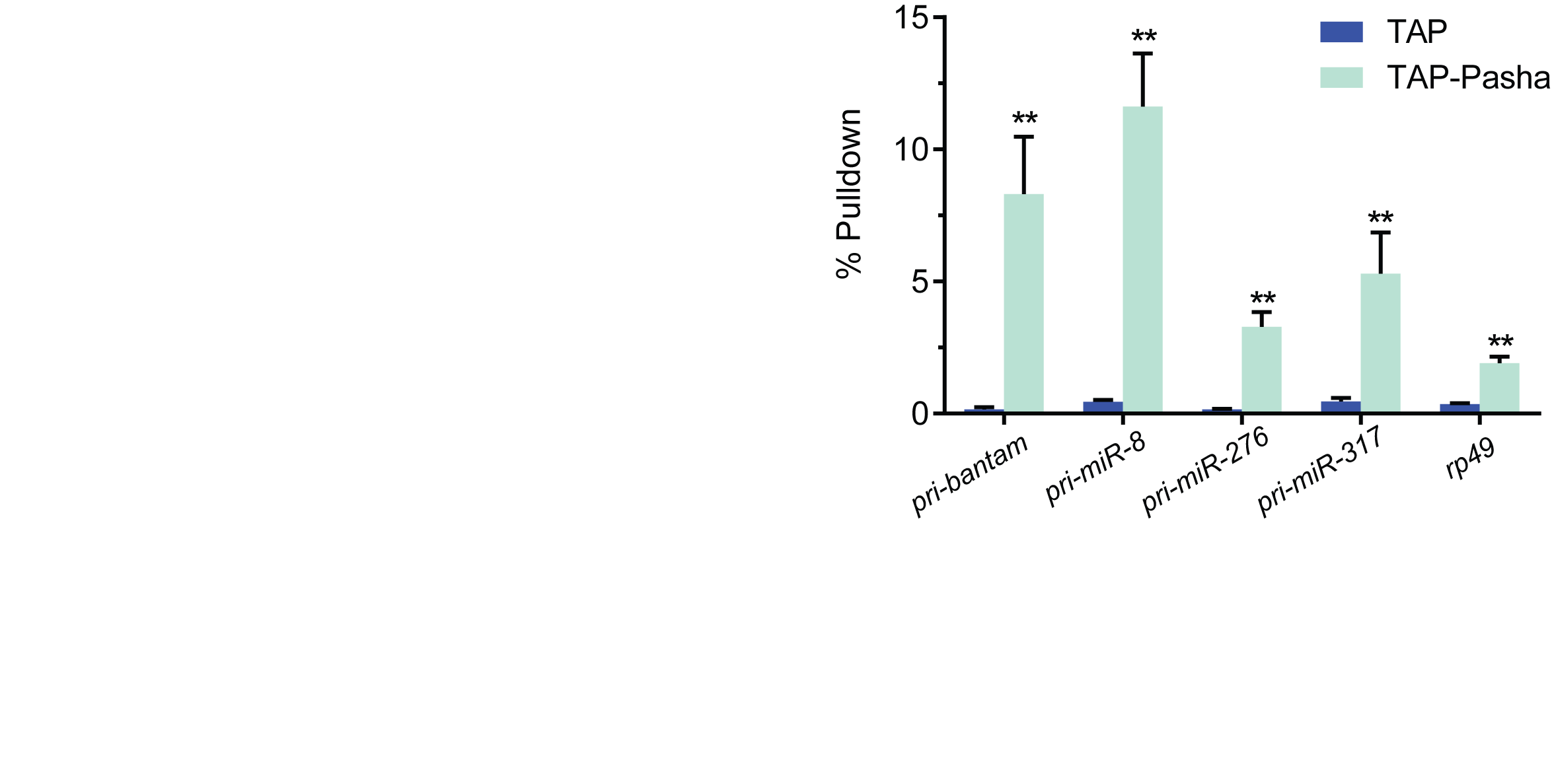

Supplement: S6 Fig — Total RNA was extracted from immunopurified TAP-Pasha or control samples (TAP) and subject to RT-qPCR to measure levels of various pri-miRNAs. Percentage of enrichment relative to the input samples are shown (n = 4; **p < 0.01). (TIF) [file pgen.1005475.s006.tif]

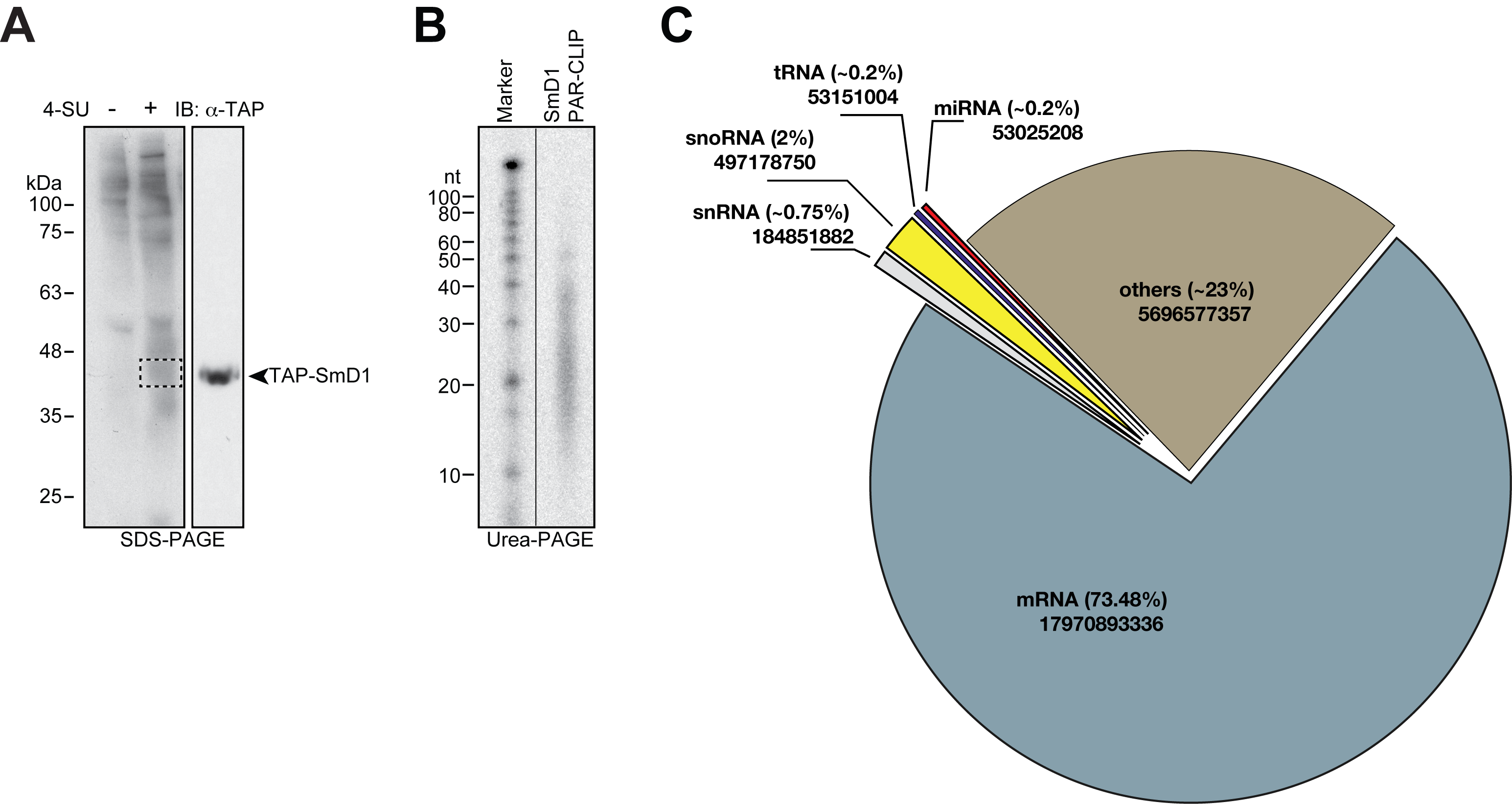

Supplement: S7 Fig — (A) S2 cells expressing TAP-SmD1 were cultured in the presence or absence of 4-thio-uridine (4-SU), and irradiated with 365 nm UV. Crosslinked protein-RNA complexes were immunopurified using IgG agarose, treated with RNase T1 to fragment RNAs, radiolabeled with T4 polynucleotide kinase, subject to SDS-PAGE, transferred to nitrocellulose membrane and visualized by phosphorimager. An immunoblot (IB) of the non-crosslinked TAP-SmD1 is shown on the right. (B) RNA was extracted from membrane slices (marked by a dashed rectangle in A), subject to 6% Urea-PAGE, and detected by autoradiography. (C) A piechart showing raw read counts and corresponding percentages of mapped reads derived from various classes of RNAs. (TIF) [file pgen.1005475.s007.tif]

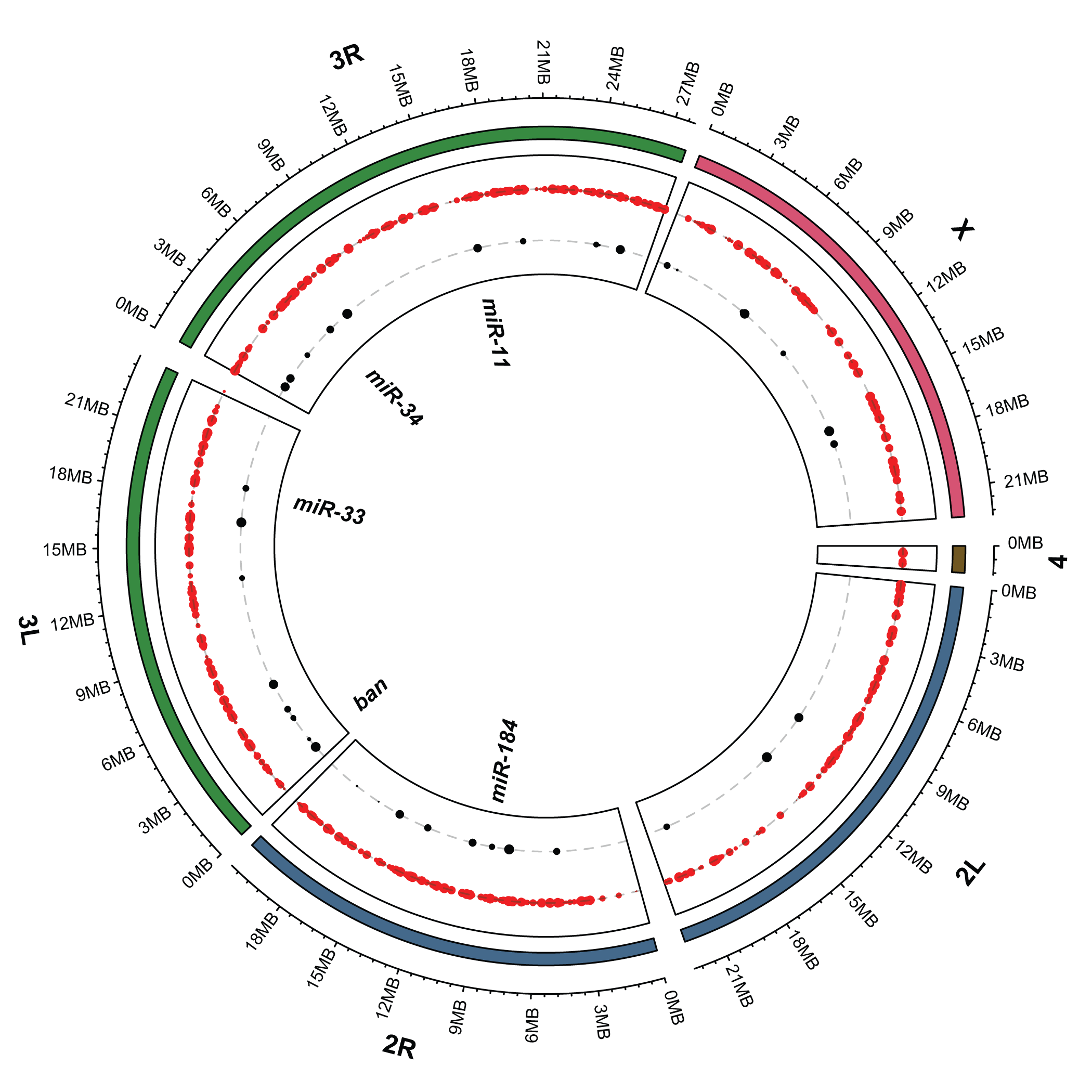

Supplement: S8 Fig — Scaled curves at the outer circle indicate various chromosomes, whereas clusters mapped to the coding and non-coding RNAs are illustrated with filled circles in red and black, respectively. The size of the filled circles reflects the modeScore value for every cluster [score of the highest signal / (signal + background)] value generated by PARalyzer. Select miRNAs are indicated inside the circle. (TIF) [file pgen.1005475.s008.tif]

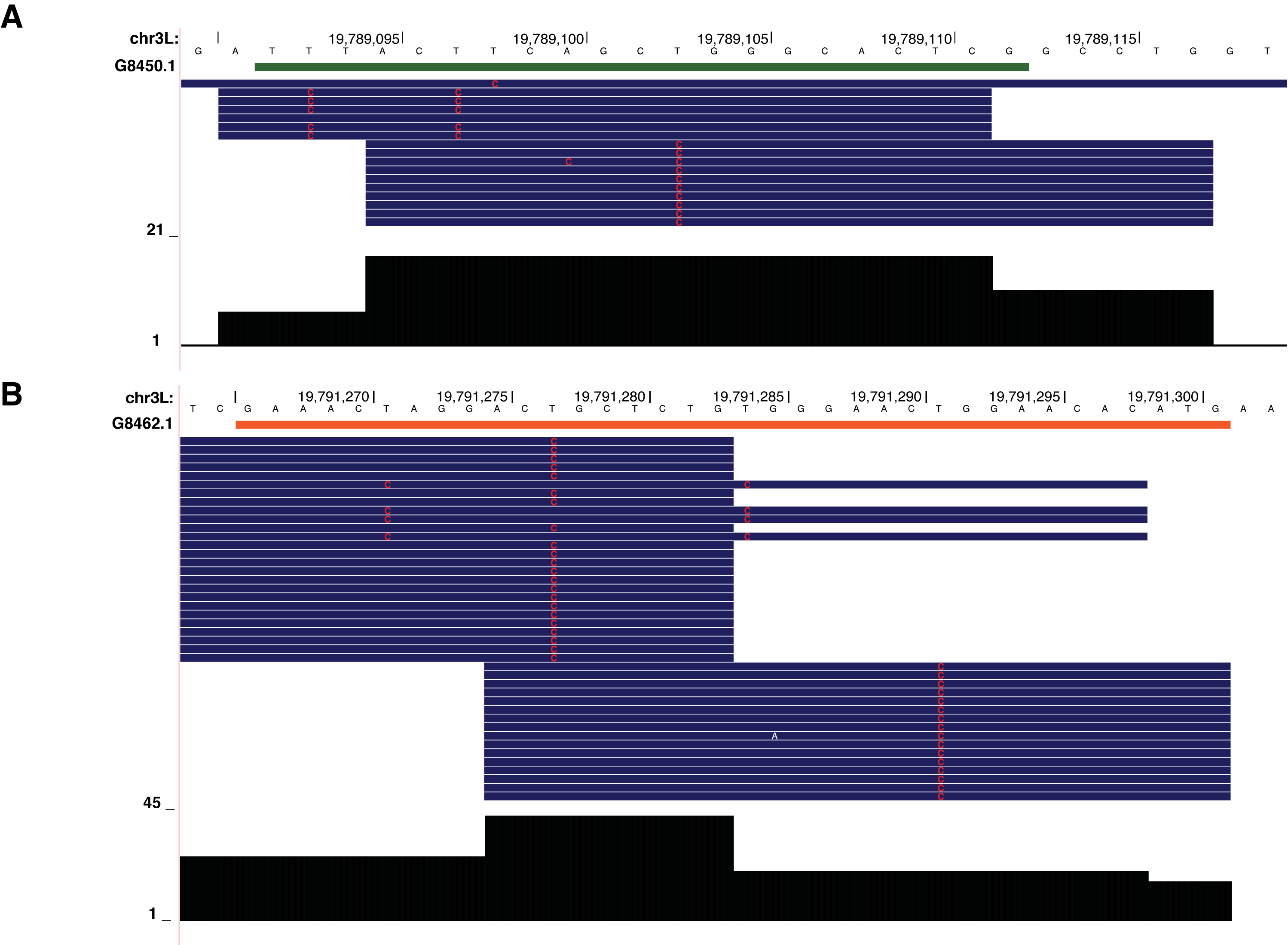

Supplement: S9 Fig — A and B illustrate two SmD1-bound clusters at the miR-33 locus, corresponding read coverage and T to C conversion events are shown. (TIF) [file pgen.1005475.s009.tif]

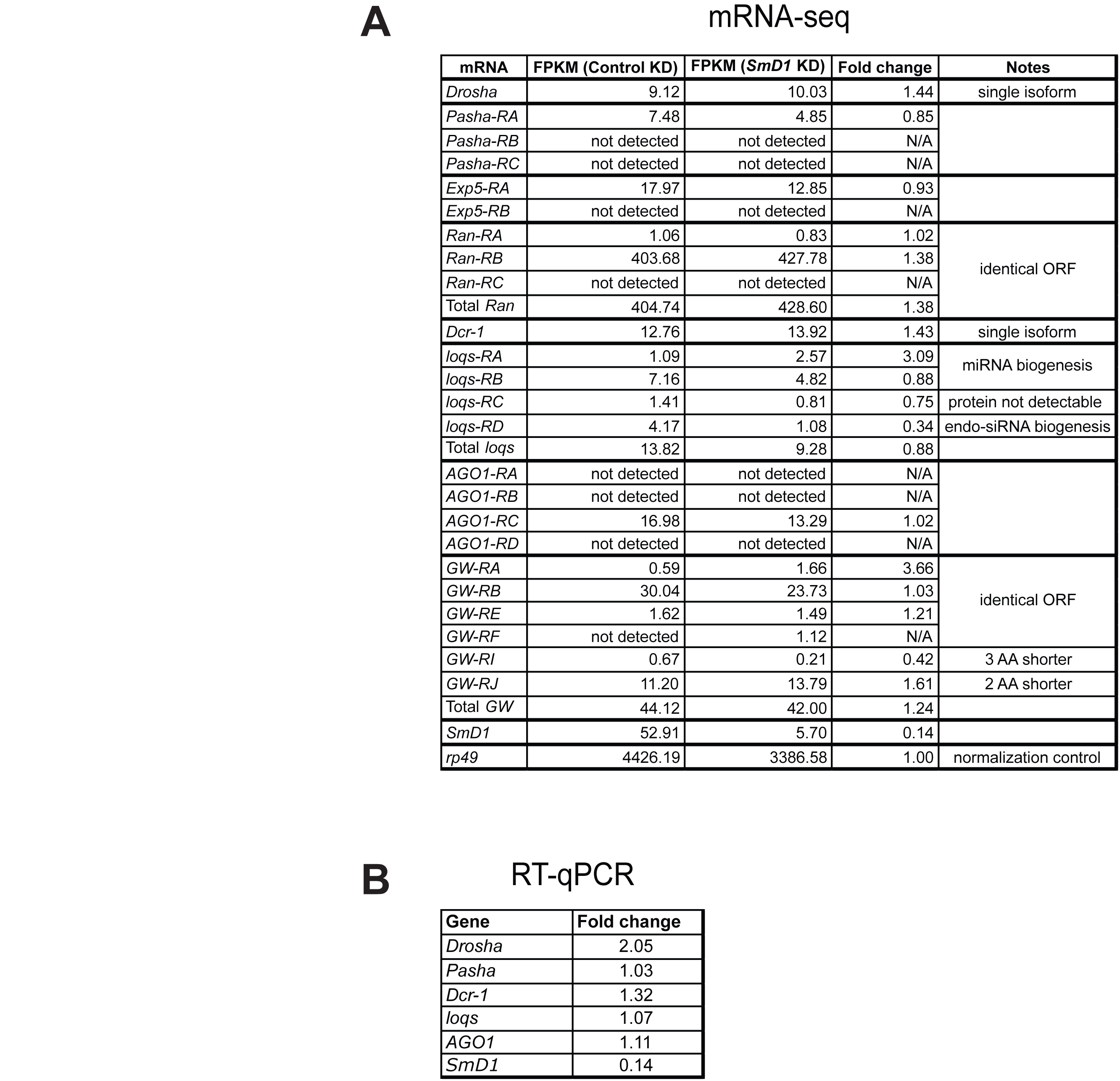

Supplement: S10 Fig — (A) RNA samples from SmD1 knockdown cells or controls cells were subject to RNA sequencing. Read counts for various splice variants of canonical miRNA pathway components are shown. Upon normalization to the control rp49 mRNA, fold changes in mRNA levels of canonical miRNA factors in SmD1-depleted cells relative to control samples are calculated. (B) RT-qPCR was employed to validate the deep sequencing results from A. (TIF) [file pgen.1005475.s010.tif]

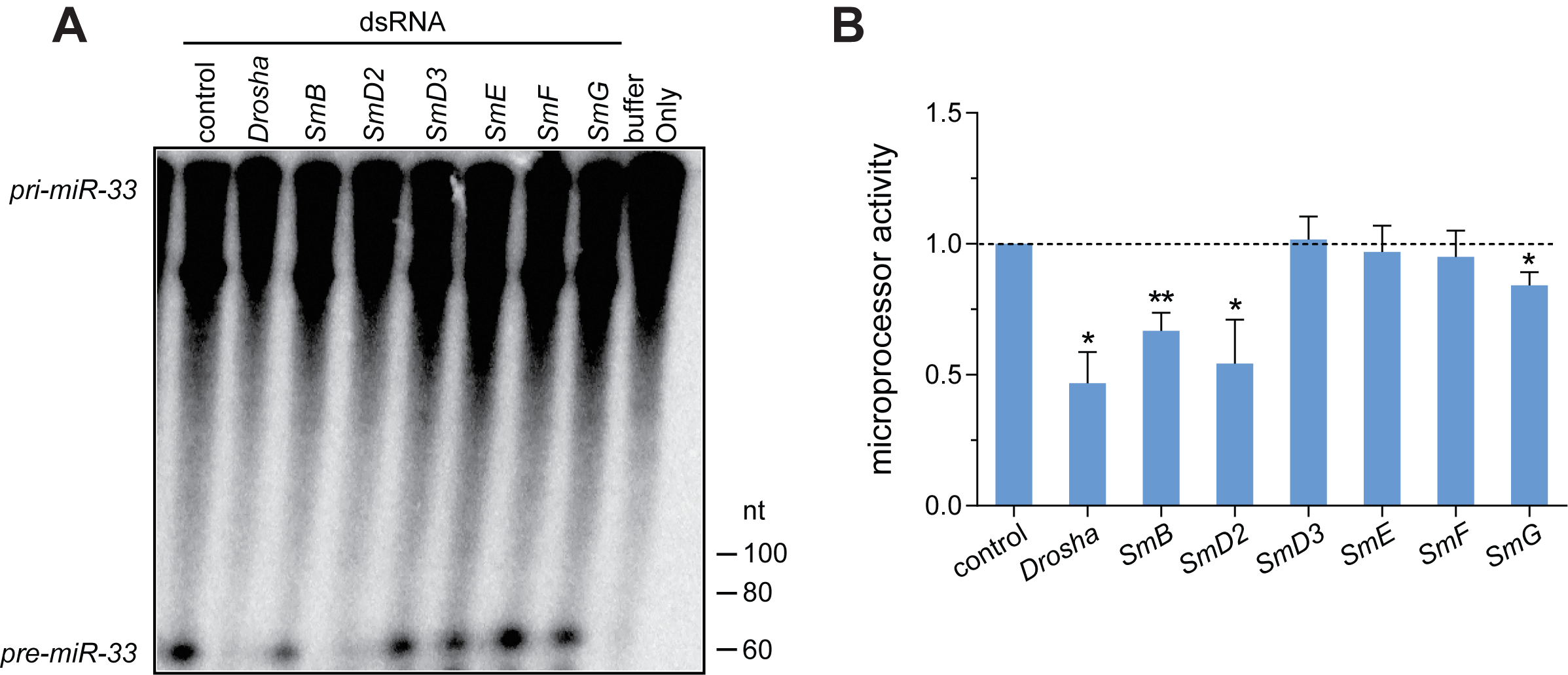

Supplement: S11 Fig — (A,B) Microprocessor activities in lysates from various dsRNA-treated cells (top) were assayed in A and quantification results are shown in B (n ≥ 3; mean + SEM; **p < 0.01). (TIF) [file pgen.1005475.s011.tif]

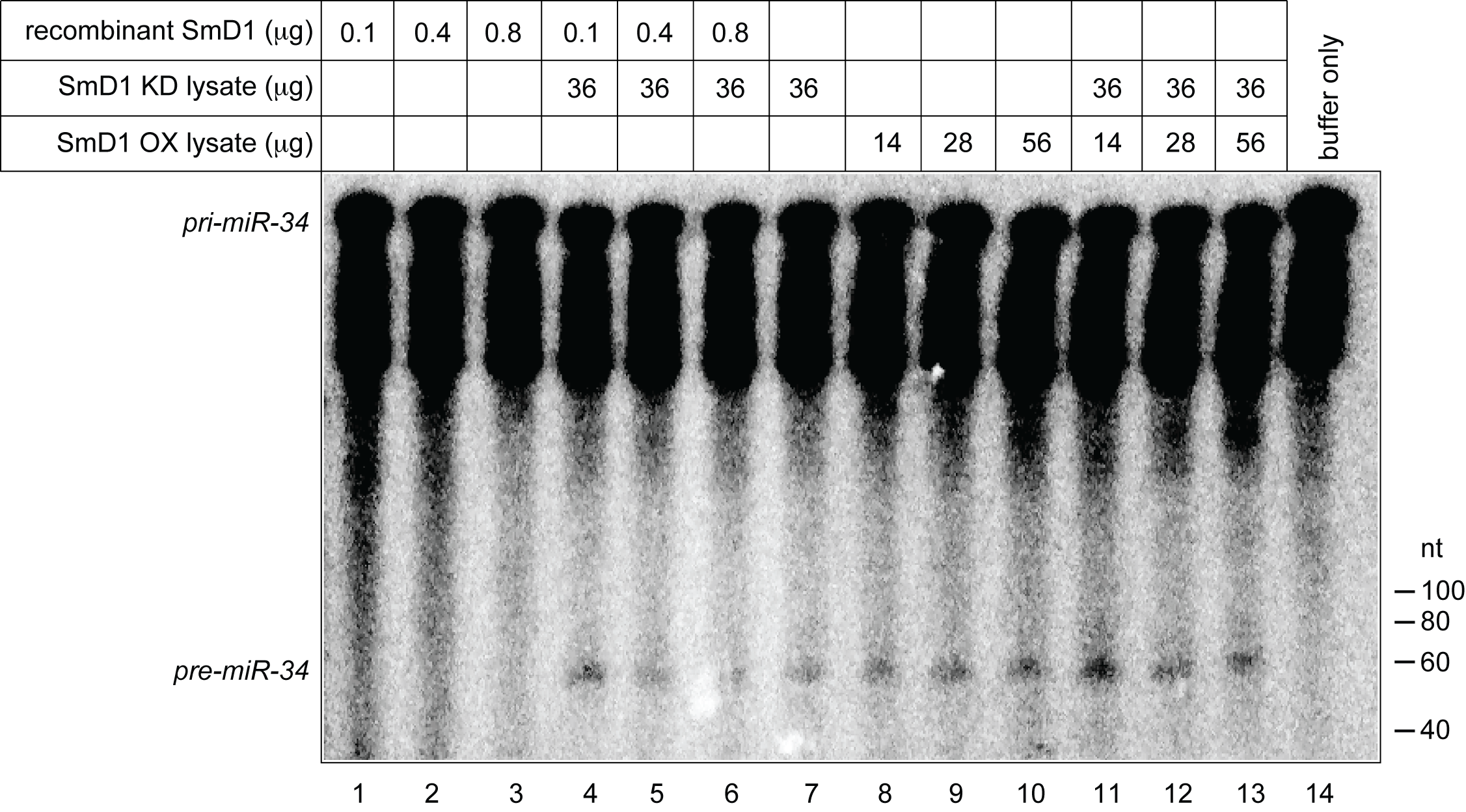

Supplement: S12 Fig — Various combinations of recombinant SmD1 or lysates from SmD1-overexpression cells or SmD1-knockdown cells were subject to microprocessor assay using pri-miR-34 as substrate. (TIF) [file pgen.1005475.s012.tif]

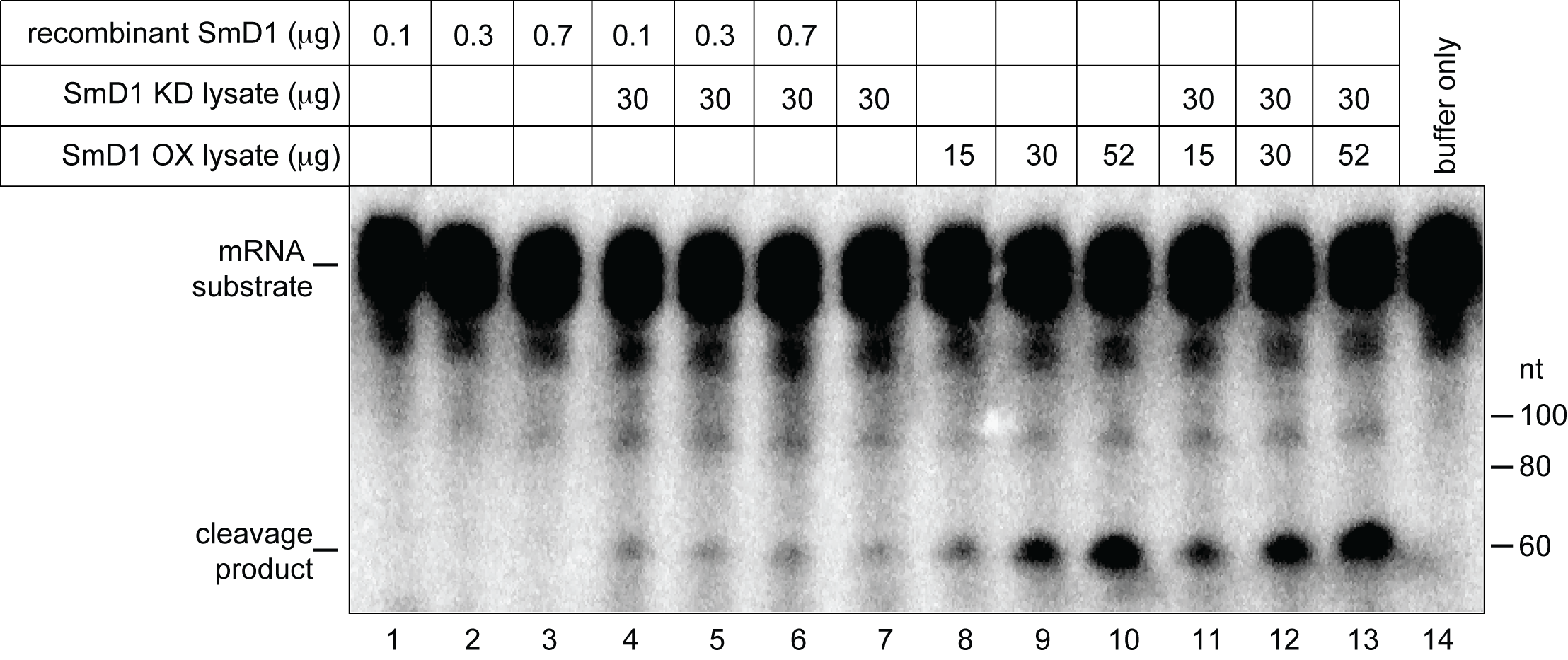

Supplement: S13 Fig — Various combinations of recombinant SmD1 or lysates from SmD1-overexpression cells or SmD1-knockdown cells were subject to AGO1-miRISC slicing assay as described in 4F. (TIF) [file pgen.1005475.s013.tif]
